# Rare-Earth Silicates as High-Temperature Surfactants for the Controlled Synthesis of ε‑Fe_2_O_3_ Nanoparticles

**DOI:** 10.1021/jacs.5c05058

**Published:** 2025-09-04

**Authors:** Naureen Khanam, Zheng Ma, Sergi Ortiz Ropero, Nico Dix, Ana Vila Costa, Judit Oró-Solé, José Luis García-Muñoz, Jordi Faraudo, Martí Gich

**Affiliations:** Institut de Ciència de Materials de Barcelona, ICMAB-CSIC, carrer dels Til·lers, Cerdanyola del Vallès, 08193 Barcelona, Spain

## Abstract

The functional properties of nanocrystals can be finely tuned through controlled morphology and size. However, this can be challenging for metastable nanostructures that require harsh synthesis conditions, such as high temperatures. Here, we present a method for preparing large ε-Fe_2_O_3_ nanorods that are not affected by magnetic relaxation. This study presents a novel growth mechanism in which high-aspect-ratio rods evolve from spherical ε-Fe_2_O_3_ particles in a silica matrix containing Y^3+^. With the presence of Y^3+^, the glassy matrix undergoes a metastable binodal decomposition yielding the formation of nanodroplets of a Y-rich silicate of composition ∼Y_2_Si_2_O_7_. This Y silicate selectively coats the ε-Fe_2_O_3_ planes perpendicular to the rod axis along the [100] direction but is not observed in the rod apexes. Structural optimizations and energy calculations of different crystal faces of ε-Fe_2_O_3_ in contact with Y_2_Si_2_O_7_ obtained using machine-learning force fields provide an atomistic interpretation of these observations: the affinity of Y with the oxygen atoms exposed at ε-Fe_2_O_3_ surfaces explains the preferential capping of ε-Fe_2_O_3_ surfaces that present a large density of oxygen atoms and its absence in surfaces such as (100), where this density is significantly lower. The presence or absence of the silicate capping layer results in different surface energies and/or mass transfer coefficients across the interface, originating two independent Ostwald ripening processes, which drive the high aspect ratio growth. By using La^3+^ instead of Y^3+^, ε-Fe_2_O_3_ rods with even larger aspect ratios are obtained. Notably, this synthetic approach counteracts the progressive diminution of the average nanoparticle size observed in ε-(Fe_1–*x*_Cr_*x*_)_2_O_3_ upon Cr^3+^ addition, enabling to elucidate the effect of this substitution on the intrinsic magnetic anisotropy and the anisotropy fields that determine the high-frequency ferromagnetic resonances of this phase.

## Graphical abstract

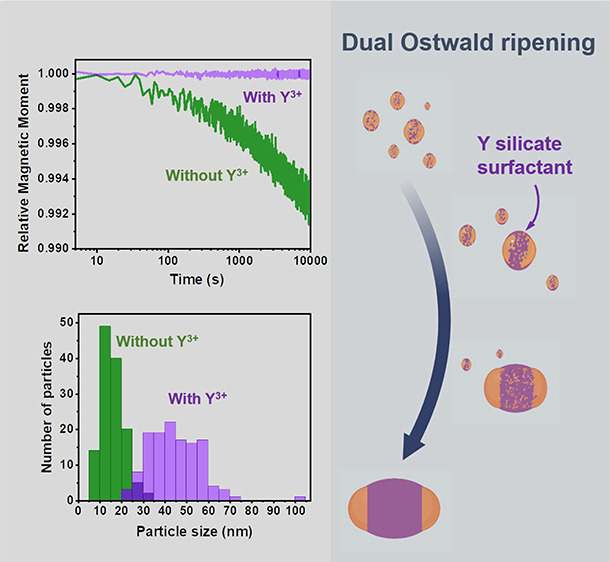

## Introduction

The size and shape of nanocrystals can significantly influence many of their properties, from those considered intrinsic to the crystalline phase to those related to the material’s interactions with external fields and the surrounding media. For instance, by decreasing nanoparticle size, melting can be observed at lower temperatures,^1^ or ferromagnetic systems can display size-dependent responses to magnetic fields such as superparamagnetism.^2^ The relevance of nanocrystal shapes is well illustrated by how the aspect ratio of metal nanoparticles determines their surface plasmon resonance frequencies,^3^ or by the critical dependence of the nanocatalyst responses on the specific exposed facets.^4^ Thus, controlling the size and shape of nanocrystals is crucial for tailoring their functional properties and constitutes a cornerstone of their development for applications. A common strategy is adjusting parameters governing nanocrystals’ nucleation and growth rates, such as temperature, reaction times, the concentrations of reagents, type of solvents, or the pH, which allows tuning the solution’s ionic strength to modify the surface charges of crystals.^5,6^ Indeed, modifying the interfacial energy between the forming nanocrystals and the surrounding medium is also a key strategy for controlling their morphologies. A broad range of additives, from amphiphilic molecules^7^ and other surface-binding ligands to complexing agents^8^ or solid particles,^9^ as well as templates, can play the role of surface-active agents (surfactants). These principles have underpinned substantial progress in nanocrystallization, and biomineralization, with its genome-encoded protocols, illustrating their potential to produce structures with an extraordinary degree of control over the nanocrystal morphologies and their oriented assembly.

While most of these developments involve solution synthesis at mild temperatures, the formation of technologically relevant nanomaterials can require high temperatures that are not compatible with standard solvents and surfactants, making the control of nanocrystal morphologies more challenging. Moreover, at high temperatures, the growth of particles tends to be governed by Ostwald ripening, which promotes the growth of large crystals at the expense of the small ones. Under this mechanism, the control of the crystal morphologies through the temperature and times of annealing can be rather limited. This is because the growth of nanocrystals is often driven by the minimization of surface energies and largely determined by intrinsic material characteristics, such as diffusion coefficients and interfacial energies.

These limitations can make it challenging to tune the functional properties of unique materials intended for specific applications. This is the case of ε-Fe_2_O_3_, a metastable polymorph of ferric oxide prepared at 1100 °C,^10,11^ with a large magnetic anisotropy^12,13^ that displays a very high natural ferromagnetic resonance which makes it appealing for the millimeter wave-assisted switching of the magnetization^14^ and applications in the field of millimeter waves, such as in stealth technologies and nonreciprocal devices for wireless communications.^15–17^ However, the sizes of ε-Fe_2_O_3_ nanoparticles that can be achieved with scalable production methods are too small to retain the magnetization for a long time. Even more, as we will see, the size limitation can conceal the real effect of cationic substitutions on intrinsic magnetic properties of relevance to technological applications. To overcome these issues, in this work, we propose a new approach to prepare ε-Fe_2_O_3_ nanoparticles coated with rare earth silicates that act as high-temperature surfactants, altering the Ostwald ripening and facilitating further nanoparticle growth. This approach made it possible to disclose that, contrary to what had been reported, the Cr^3+^ substitution increases the magnetic anisotropy field in ε-Fe_2_O_3_, shifting the mm-wave ferromagnetic resonance to higher frequencies.

## Results and Discussion

The preparation of ε-Fe_2_O_3_ can be scaled up with a sol–gel method, which we will refer to as “bulk sol–gel” (see details in Section 1, Supporting Information (SI)) based on the thermal transformation of a bulk silica gel obtained from the hydrolysis and condensation of tetraethoxysilane (TEOS) in a hydroethanolic solution of iron nitrate.^13^ This approach allows obtaining in the lab several grams of roughly spherical ε-Fe_2_O_3_ nanoparticles per batch, but their average sizes do not exceed ∼25 nm for 3 h treatments at 1100 °C, the highest temperature that allows stabilizing ε-Fe_2_O_3_ as the major polymorph.^10,13^ This limited size is a drawback for the mentioned applications, for which it is crucial to retain the nanoparticles’ magnetization and avoid superparamagnetic relaxation. Indeed, from time-dependent measurements of the magnetic remanence of ε-Fe_2_O_3_ nanoparticles prepared by different methods, it is seen that magnetic relaxation critically depends on the size (Figure S3, SI). The BSG and RMSG labels in the samples of Figure S3 indicate their preparation following the bulk sol–gel or the reverse micelle methods, respectively. Note that in the BSG series, even the samples with the largest average sizes present some degree of magnetic relaxation. A comparison between the BSG_1100 and RMSG_Ba_0.1_1000 samples in Figure S3 shows that the magnetic relaxation issue of the BSG samples can be circumvented by increasing the size of ε-Fe_2_O_3_ nanoparticles with other sol–gel routes such as the RMSG. These methods also use TEOS and iron nitrate but incorporate a surfactant, hexadecyltrimethylammonium bromide (CTAB) and Ba^2+^ in the sol, either by a reverse micelle method^12,18^ or in films prepared by dip-coating.^19^ Unfortunately, these syntheses make intensive use of organics and are thus not convenient for large-scale production. For instance, while the bulk sol–gel method requires around 15 g of organic solvents per g of ε-Fe_2_O_3_,^18^ this figure increases considerably to more than 500 g for the reverse micelle method. To overcome this limitation, next we explore an alternative route to prepare significantly larger ε-Fe_2_O_3_ nanoparticles by the bulk sol–gel approach.

Since the crystallization of ε-Fe_2_O_3_ occurs at temperatures well above the decomposition of CTAB, we turned our attention to the specific role of Ba^2+^ in promoting the growth of large, high aspect ratio ε-Fe_2_O_3_ nanoparticles. It has already been observed that the presence of Ba^2+^ or Sr^2+^ in the silica matrix results in the formation of a capping layer on the ε-Fe_2_O_3_ particles while growing.^20^ Those large cations diffuse in the silica matrix at high temperatures and are presumed to be adsorbed on the low-energy facets of ε-Fe_2_O_3_, directing the growth along specific orientations. However, this process is still poorly understood, in contrast to the ion-mediated anisotropic growth of metals^21–24^ and metal oxides^25,26^ in solution.

### Modified Bulk Sol–Gel Method to Increase Nanoparticle Sizes

Given the poor solubility of common Ba salts in hydroethanolic solutions, we initially soaked as-prepared silica/iron nitrate gels in 6 mM aqueous solutions of Ba^2+^ for 20 h to incorporate the cation. Then, the gels were dried (60 °C, 24 h) and annealed (3 h at 1100 °C in air, heating ramp 80 °C/h). The resulting silica/ε-Fe_2_O_3_ composite (BSG_Ba_1100) presented, compared to the reference (BSG_1100), larger particle sizes and aspect ratios, and the remanent magnetization remains more stable (see S3a,b). Nevertheless, this approach was highly nonreproducible. In parallel, we observed the crystallization of silica in reference samples prepared by the reverse micelle method^18^ with different Ba^2+^ contents, even at annealing temperatures of 1000 °C (see S3d). This is an unwanted consequence of the marked glass modifier character of a cation with such low electronegativity and cationic field strength. In practice, this makes the use of Ba^2+^ unsuitable for upscaling the ε-Fe_2_O_3_ production by the bulk sol–gel method because silica devitrification triggers the appearance of significant amounts of hematite impurities in silica/ε-Fe_2_O_3_ composites.^10^ These preliminary trials highlight the need to explore other cations that can also induce the growth of large high aspect ratio ε-Fe_2_O_3_ nanoparticles but without interfering with the stability of the silica glass. For this, we selected cations that (i) are too large to be incorporated into the iron oxide structure, (ii) are not prone to promote the crystallization of the silica glass and (iii) have highly soluble precursors in hydroethanolic media. The first condition is met by cations of radii, *r*_c_, that do not fulfill the first Pauling rule for ionic oxide structures with tetrahedral and octahedral coordination, given by $0.225<\frac{r_{c}}{r_{O^{2-}}}<0.645$ , where the oxygen radius *r*_O^2–^_ is about 140 pm. The second constraint is motivated by the effect of some cations to significantly lower glass transition temperatures and favor glass crystallization. The cationic field strength, *A*, defined as the ratio of the cation valence *Z*_c_ to the square of the minimum cation–oxygen distance in Å, $\frac{Z_{c}}{(r_{c}+r_{O^{2-}}{)}^{2}}$ was proposed by Dietzel^27^ to classify the role of the cations in oxide glasses between glass network formers (when *A* > 1 |*e*|·Å^–2^) and glass network modifiers, which are more likely to promote crystallization (if *A* < 0.35 |*e*|·Å^–2^). Thus, to satisfy criterion (ii), we considered cations with intermediate field strengths (0.35 < *A* < 1 |*e*|·Å^–2^). The selection criteria (i) to (iii) are fulfilled by Y^3+^ and trivalent cations of the lanthanide series.

Next, using a modified bulk sol–gel synthesis that incorporates Y nitrate in the initial sol, we prepared a series of ε-Fe_2_O_3_ with various relative molar concentrations of Y^3+^ with respect to the total molar concentration of metal cations (i.e., *x* = [Y^3+^]/([Y^3+^] + [Fe^3+^]) (see details in Section S1 and Table S2). The samples obtained from the dried gels annealed for 3 h at 1100 °C were labeled as *Yx*, where *x* stands for the yttrium concentration *x* multiplied by 100. The Rietveld analysis of X-ray diffraction (XRD) patterns (SI, Figure S4 and Table S3) confirms that ε-Fe_2_O_3_ is the major phase in these samples. The crystallographic unit cell volume remains constant as increasing amounts of Y^3+^ are added to the solutions, corroborating that this cation is not substituting Fe^3+^ in the ε-Fe_2_O_3_ structure. These results indicate that the introduction of Y^3+^ in the synthesis makes it possible to reproducibly prepare significantly larger and rod-shaped ε-Fe_2_O_3_ nanoparticles, not affected by magnetic relaxation issues. This is clearly evidenced in Figure 1 with the representative low-resolution transmission electron microscopy (TEM) images and volume and aspect ratio distributions of ε-Fe_2_O_3_ nanoparticles obtained from samples Y1 (Figure 1a,c) and Y10 (Figure 1b,d) after etching the SiO_2_ matrix. The analogous data for the samples Y3, Y5, Y15, and Y20 are presented in Figure S5.

**Figure 1:**
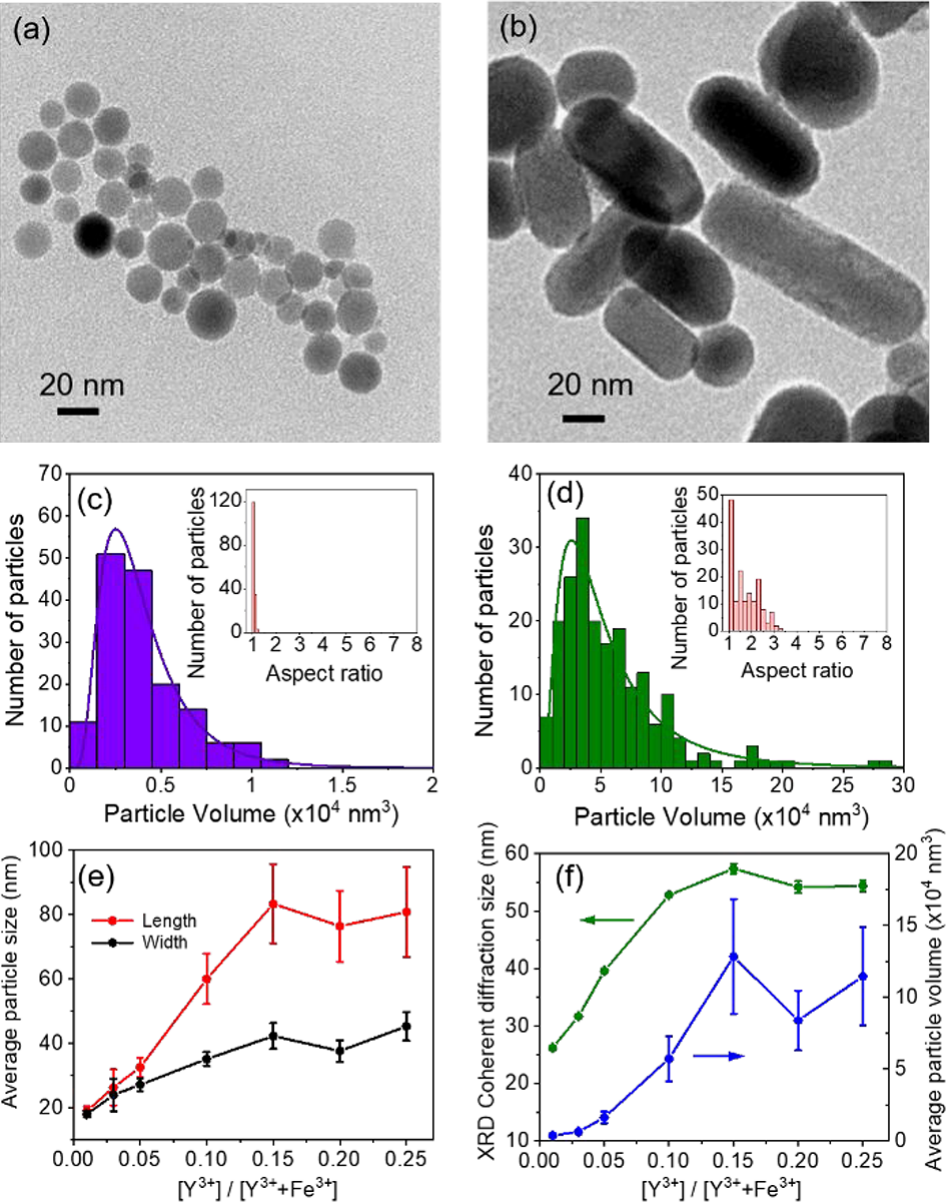
(a, b) Representative TEM images of ε-Fe_2_O_3_ nanoparticles prepared with different Y^3+^ content *x* = 0.01 and *x* = 0.1, corresponding to samples Y1 and Y10, respectively; (c, d) particle volume distributions and aspect ratios (inset) obtained from analyzing TEM images of the same samples displayed in (a) and (b), respectively; (e) evolution of the average particle dimensions (length and width) obtained from TEM as a function of the relative molar Y^3+^ content with respect to Fe^3+^; and (f) evolution of the coherent diffraction size obtained from Rietveld refinement and average particle volume as a function of the relative molar Y^3+^ content with respect of Fe^3+^.

The average characteristic sizes of the particles (lengths and widths) obtained both from TEM images (Figure 1e), the average values obtained of the volume distributions and diffraction crystal size obtained from XRD line broadening (Figure 1f) steadily increase with the Y^3+^ content up to 15 at % (*x* = 0.15). For instance, the crystal size determined from XRD for the *x* = 0.1 sample doubles the value of the sample with the lowest Y^3+^ content (*x* = 0.01), and a maximum size is reached for the *x* = 0.15 sample. This increase tends to level off for higher Y^3+^ content. From Figure 1e one can also notice that while up to *x* = 0.05 the particle lengths are only slightly larger than its widths, for *x* = 0.1 the aspect ratio undergoes a noticeable jump, suggesting the existence of a specific concentration threshold for the growth of nanorods at this Y^3+^ concentration.

To shed more light on the Y^3+^ in the growth of nanorods, a scanning transmission electron microscopy (STEM) study was conducted on selected samples before and after etching the silica. Figure 2a presents in the top left corner a Z-contrast image of a piece of Fe_2_O_3_/SiO_2_ composite corresponding to sample Y15 (*x* = 0.15), acquired with a high angle annular dark field detector (HAADF) before etching the silica matrix.

**Figure 2:**
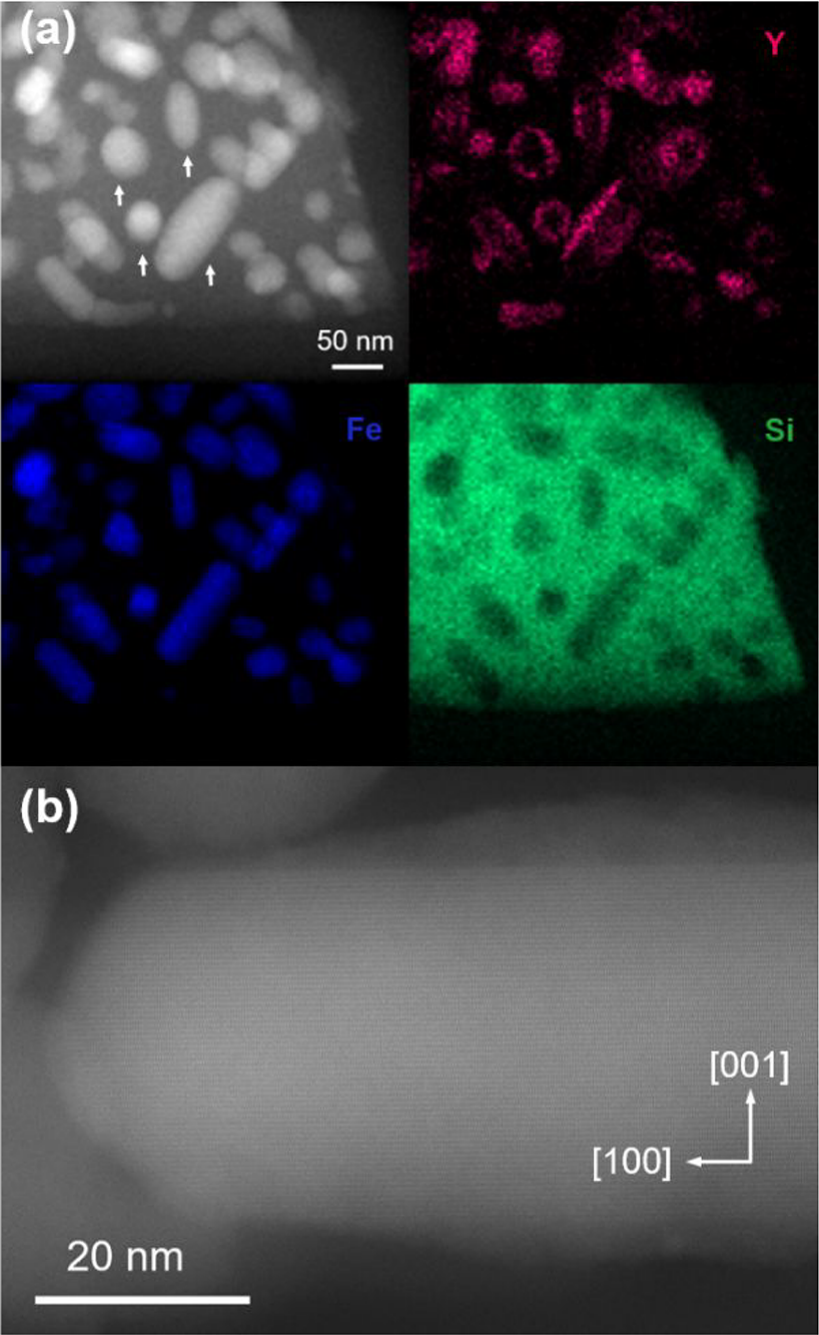
(a) STEM-HAADF image and Y, Fe, and Si elemental maps collected from EDX of a group of ε-Fe_2_O_3_ nanoparticles of sample Y15, prepared with a Y concentration *x* = 0.15. The white arrows in the HAADF image indicate the specific particles mentioned in the figure description in the manuscript. (b) high-resolution STEM-HAADF image of a single nanorod corresponding to the same sample after etching the silica matrix in a view along the [010] zone axis.

The corresponding elemental distribution maps of Y, Fe, and Si, acquired by energy dispersive X-rays (EDX) analysis, are presented in the other panels in magenta, blue and green, respectively. The location of the iron oxide particles embedded in the silica matrix can be clearly distinguished both in the Z-contrast image and the Fe and Si elemental maps of Figure 2a, while O is present in all the material (see S6). Interestingly, while there are nearly spherical particles, some particles are elongated and exhibit the shape of a prolate ellipsoid (see for instance those marked by vertical arrows in the Z-contrast image). Moreover, for the elongated particles, Y is mostly present at the particle sides along the axes of the ellipsoids and less concentrated in the central part of the particle, but we can notice that almost no Y is observed at their apexes. In contrast, for the round particles, Y is distributed all around the particle, defining a circle around the particle boundary with the silica matrix, but no Y is found in the inner part of this circle. Regarding the Fe and Si concentrations, the Fe distribution defines a perfectly straight rod with parabolic apexes instead of the prolate ellipsoid of the Z-contrast image, with the particularity that Si is absent in the zones occupied by Fe. For the rounded particles, Fe is found (and Si is not present) at the inner part of the circles defined by Y, also occupying, in these cases, a smaller area than that of the light contrast zone in the Z-contrast image. These observations suggest that Y is only present around the ε-Fe_2_O_3_ rods, all along their axis except at the apex, and that the rounded particles correspond indeed to rods with their axis perpendicular to the image plane. One can also conclude that Si is present in the zones occupied by Y. Note that the EDX analysis of the same Y15 sample after etching the silica also presents Y and Si, together with O, all around the ε-Fe_2_O_3_ rods except at their ends (see S7). This indicates the existence of a Y silicate coating that has not been etched out with the silica. Figure 2b is a representative atomic-resolution Z-contrast view of the ε-Fe_2_O_3_ rods of the etched Y15 sample along the ε-Fe_2_O_3_ [010] zone axis. The rod axis is oriented along the [100] direction of ε-Fe_2_O_3_, as deduced from indexing the fast Fourier transform (FFT) pattern of the atomic resolution image (see S8). Remarkably, there is a sharp interface between the ε-Fe_2_O_3_ rod and a shell in which no crystallographic ordering can be observed. Selected area electron diffraction of the nanorod and the Y silicate shell only showed diffraction peaks that can be ascribed to ε-Fe_2_O_3_, suggesting that the Y silicate layer is amorphous (see S9). The shell thickness is maximal at the central part of the rod and progressively decreases along the axis, vanishing before reaching the apexes. The compositional analysis of this shell by EDX indicated the sole presence of Y, Si and O in a stoichiometry with equal atomic % of Y and Si (S10). In specific zones of sample Y15 one could find large accumulations of partially crystalline Y silicate with the Y_2_Si_2_O_7_ stoichiometry in contact with ε-Fe_2_O_3_ (see Figure S11, Tables S4, and S5). With the FFT of the atomic resolution image, it was identified as the low-temperature polymorph y-Y_2_Si_2_O_7_ (space group *P*2_1_/*m*).^28^ However, Y_2_Si_2_O_7_ presents 6 polymorphs^29^ and indeed, the XRD analysis of sample Y25, with the largest Y concentration, revealed the presence of β-Y_2_Si_2_O_7_ another monoclinic polymorph (space group *P*2_1_/*c*) (see S4). The appearance of Y_2_Si_2_O_7_ is expected at the SiO_2_-rich side of Y_2_O_3_–SiO_2_ phase diagram,^30^ next to the miscibility gap that exists for the compositional range of our materials (0.002 < Y:Si < 0.06). Indeed, Y_2_Si_2_O_7_ nanospheres were reported in silica materials with Y prepared by sol–gel (using Y:Si = 0.1) after annealing at 1100 °C.^31^ We prepared a silica gel without iron nitrate with the same pH and Y:Si molar ratio as in the synthesis of sample Y10, and the dried gel was also annealed for 3 h at 1100 °C. The resulting material consists of nanodroplets (diameter ∼20 nm) of amorphous Y silicate of composition ∼Y_2_Si_2_O_7_ embedded in a SiO_2_ glass (see S12). The existence of this metastable binodal decomposition^32^ appears as a first necessary condition for the action of Y silicate as surface active agent. A second requisite is a strong affinity of this Y-rich glassy phase for the crystalline precipitates in the silica matrix. Indeed, we observed, a marked tendency of Y silicate to be in contact with ε-Fe_2_O_3_ instead of just forming droplets within the silica, suggesting that the ε-Fe_2_O_3_–Y_2_Si_2_O_7_ interfacial energy is lower than that of SiO_2_–Y_2_Si_2_O_7_. Since the phase separation tendency correlates with the cationic field strength, a similar behavior can be expected in other metal silicate systems.^33^

To test the versatility of the method and investigate specific influences of the different rare earth (RE) cations, we prepared ε-Fe_2_O_3_/SiO_2_ composites with different concentrations of Ce^3+^, Dy^3+^, and La^3+^. In the case of Ce^3+^, CeO_2_ was formed, and thus we did not observe any significant increase in the size of ε-Fe_2_O_3_ nanoparticles. In contrast, Dy^3+^ and La^3+^ had an impact on the growth of ε-Fe_2_O_3_ nanoparticles analogous to that of Y^3+^ but attaining, particularly with La^3+^, larger rod lengths, aspect ratios and overall crystal sizes (see S13–S15). This is clearly evidenced in Figure 3a, which compares the distributions of aspect ratios of ε-Fe_2_O_3_ nanoparticles obtained with La^3+^ and Y^3+^, as well as with their widths and lengths, presented in the inset.

**Figure 3:**
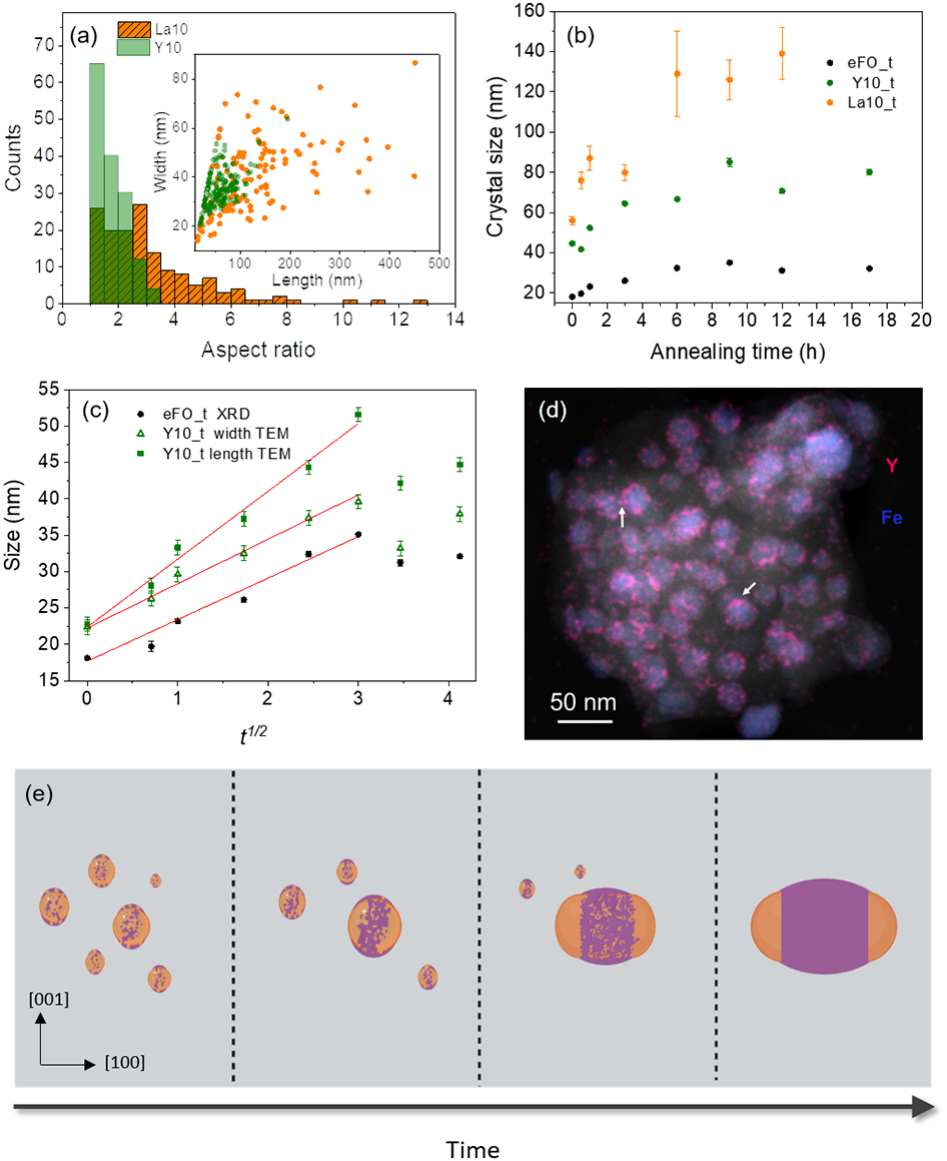
(a) Aspect ratio distribution of ε-Fe_2_O_3_ nanoparticles prepared with La^3+^ (*x* = 0.1) and Y^3+^ (*x* = 0.1) corresponding to samples La10 (orange) and Y10 (light green), respectively. The dark green shaded area corresponds to the overlap of the distributions. The inset is a plot of the width vs length of the sampled nanoparticles from both samples; (b) time-dependent average crystal sizes obtained from Rietveld refinement of ε-Fe_2_O_3_ nanoparticles prepared with Y^3+^ (Y10), La^3+^ (La10), and without rare earth additives (eFO_*t*); (c) evolution of the size of ε-Fe_2_O_3_ nanoparticles as a function of the square root of annealing time at 1100 °C. The red lines are linear fits to the data. For ε-Fe_2_O_3_ prepared without rare earths, sizes were obtained from the XRD Rietveld refinement (eFO_*t* XRD). For the sample prepared with Y^3+^, the sizes correspond to the lengths (Y10_*t* length TEM) and widths (Y10_*t* width TEM) obtained from TEM images; (d) overlap of an HAADF image and the Y and Fe elemental maps of ε-Fe_2_O_3_ nanoparticles prepared with Y^3+^ just heated to 1100 °C (Y10_0 h) and immediately quenched. The accumulation of Y on specific zones of the roughly spherical ε-Fe_2_O_3_ is indicated with arrows; (e) sketch of the Ostwald ripening process in ε-Fe_2_O_3_ nanoparticles. The Fe_2_O_3_ particles are shown in brown, the Y silicate shell on the facets parallel to the [100] direction is represented in violet, and the gray background is the silica matrix.

To further validate the hypothesis by which we established the cation selection conditions (i) to (iii) we also tested the lower limits of the condition (ii) with the effect of elements such as Bi^3+^ and Cd^3+^, which present cationic strengths of *A*_Bi_^3+^ = 0.45 |*e*|·Å^–2^, and *A*_Cd^2+^_ = 0.36 |*e*|·Å^–2^, which are slightly above the 0.35 |*e*|·Å^–2^ limit for the classification of glass modifier cations, but significantly lower than the *A* values of Y^3+^, La^3+^ or Dy^3+^, (0.57, 0.52, and 0.56 |*e*|·Å^–2^, respectively). We also tested the influence of Sb^3+^, with a high cationic strength, *A*_Sb^3+^_ = 0.66 |*e*|·Å^–2^, but with a size that allows substitution of Fe^3+^ in ε-Fe_2_O_3_. Interestingly, at relatively low concentrations (*x* = 3), these three cations already show significant enhancement of nanoparticle sizes in comparison with the equivalent concentration of Y^3+^ (see S16). However, for Bi^3+^ and Cd^3+^ the cationic strength seems to be too low, because the thermal treatments induced the silica glass crystallization and the appearance of large volumes of hematite together with nanoparticles (see XRD patterns in S17). In the case of Sb^3+^, as expected, the Rietveld refinement of XRD patterns evidenced substitution into the ε-Fe_2_O_3_ structure (Table S6).

### Impact of Annealing Time, Temperature, and Processes Governing the Growth

To better understand the role of rare earths in the growth of ε-Fe_2_O_3_ rods we prepared three new series of materials by the bulk sol–gel method and annealed them at 1100 °C in air for times ranging from 0 to 18 h (see details in Supporting Information Section 1). Two of the series were prepared with rare earths, namely Y^3+^ and La^3+^ at concentrations of *x* = 10, and the resulting materials were respectively labeled as Y10_*t* and La10_*t*, with *t* being the duration of annealing at 1100 °C in h. For comparison, we prepared a third series, labeled as eFO_*t*, according to the standard bulk sol–gel synthesis without rare earths. Figure 3b presents the increase with the annealing time of crystal sizes, obtained from the broadening of XRD pattern, showing that the growth is boosted by the presence of Y^3+^ and even more in the case of La^3+^. The size of ε-Fe_2_O_3_ crystals increases up to 9 h and then slightly decreases for eFO_*t* and Y10_*t*, while a plateau is reached at 6 h for La10_*t*.

In Figure 3c, we can see that the increase in the crystal size of eFO_*t* is proportional to *t* ^1/2^, indicating that the process is governed by interface-controlled Ostwald ripening in which the growth is not limited by diffusion but by the mass transfer rate at the particle–matrix boundary.^34^ Interestingly, the same type of dependence is observed in the Y10_*t* series for both the rod widths and lengths, taken as the mode of the distributions obtained from TEM images. In contrast, the Ostwald ripening model,^34,35^ which assumes spherical particles, cannot describe the evolution of the XRD crystal size for the Y10_*t* and La10_*t* series, as it is evidenced by the deficient linear fits of their *t* ^1/2^ plots (SI, Figure S18).

The results of Figure 3c reveal two independent ripening processes that govern the growth of Y10_*t* ε-Fe_2_O_3_ rods: (1) a fast ${\mathrm{r̅}}_{∥}(t)$ growth along the rod axis parallel to the [100] crystallographic direction that is characterized by the Y10_*t* length vs *t* ^1/2^ plot and (2) a much slower ${\mathrm{r̅}}_{⊥}(t)$ growth in the directions perpendicular to [100], associated with the Y10_*t* width vs *t* ^1/2^ plot. The slopes of the *t* ^1/2^ plots for eFO_*t* (5.7 ± 0.3) and the Y10_*t* width (6.1 ± 0.3) are coincident within the errors, but about 60% smaller than for the Y10_*t* length (9.3 ± 0.5) (see SI, Figure S19). Thus, the growth of the rod end surfaces in which Y does not accumulate (Figures 2 and S7) essentially follows the same ripening as the eFO_t samples prepared without Y. The ε-Fe_2_O_3_ surfaces where Y silicate is preferentially deposited, namely those parallel to the [100] direction, grow much faster during the annealing at high temperatures.

In Wagner’s theory for interface-controlled Ostwald ripening,^34^ the increase with time of the average particle radius r̅(t) depends on the interfacial energy σ and the rate constant *k* of mass transfer through the particle–matrix boundary through eq 1:

$$\mathrm{r̅}(t)≅\frac{8}{9}\sqrt{\frac{\sigma kc_{0}V_{m}^{2}}{RT}}t^{1/2}$$ 1

where *c*_0_ is the limit of the solubility of an infinitely large particle, *V*_m_ is the molar volume of the condensed phase, *R* is the gas constant and *T* is the absolute temperature. Due to the presence of the Y silicate capping, only σ and *k* are expected to be different for the two ripening processes parallel and perpendicular to [100], and from the ratio of the slopes of the corresponding *t* ^1/2^ plots, it follows that σ_∥_ *k*_∥_ ∼ 2.4σ_⊥_ *k*_⊥_. Thus, the fast growth of ε-Fe_2_O_3_ crystals along [100], which results in high aspect ratio particles, stems from the larger interfacial energy and/or mass transfer rate constant at the Y silicate/ ε-Fe_2_O_3_ interface. The increased growth rates and aspect ratios obtained by using La^3+^ instead of Y^3+^ imply that, in this case, σ_∥_ *k*_∥_ must be even larger and should be understood from chemical and microstructural differences between the Y silicate and La silicate capping layers. A tentative explanation could come from the lower cationic field strength of La^3+^ (*A* = 0.52 |*e*|·Å^–2^) compared to Y^3+^ (*A* = 0.57 |*e*|·Å^–2^). It is widely accepted that the larger the cationic field strength, the greater the compositional and thermal extents of the immiscibility field.^33^ However, the metastable binodal decomposition that occurs at 1100 °C in our silica prepared by sol–gel is not known and can be expected to differ from the equilibrium miscibility gaps of the equilibrium phase diagram occurring well above 1100 °C.^30,36^ The differences in cationic field strength can also have an impact on the glass structures that could be relevant in the diffusion processes that control the growth of nanoparticles. Structural studies of aluminosilicate glasses with rare earths^37,38^ have found that the La^3+^ coordination shells are less compact, more disordered and with a wider range of nearest neighbor distances than for Y^3+^, which could result in a mass transfer coefficient from the glassy La-silicate capping layer to ε-Fe_2_O_3_ that is larger than the Y-silicate one.

It is also interesting to observe the influence of temperature on the growth of particles within the silica matrix with Y^3+^ at a concentration of *x* = 10, by quenching the samples from different temperatures (800, 1000, 1100 °C) during the heating ramp. The particle size increases, and the number of particles per unit area decreases with the quenching temperature as expected for a growth process governed by Ostwald ripening (see S20). However, in all three samples one can observe a clustering of Y on the surface of Fe_2_O_3_ nanoparticles. In the Y10_*t* sample quenched from 1100 °C (Y10_0) Y is already concentrated on specific zones of their surfaces but it does not form a continuous layer and the Fe_2_O_3_ nanoparticles are roughly spherical (see the superposition of the Z-contrast image and EDX map in Figure 3d). This, together with the images of the Y10 nanorods obtained by annealing for 3h at 1100 °C (Figure 2) suggests that a sufficient coverage of the nanoparticle by the Yttrium silicate is necessary to trigger the nanorod growth. During high-temperature annealing, the Y located at the surface of the small dissolving ε-Fe_2_O_3_ particles diffuses to larger nonvanishing crystals where it will form increasingly larger aggregates over its surface. However, the nature of the binodal decomposition determines a specific composition for the Y silicate glass droplets (∼Y_2_Si_2_O_7_ in the present case), which in turn limits the volume of Y silicate available for coating ε-Fe_2_O_3_ particles for a given Y concentration *x*. One can estimate the relative surface coverage, *S*_YS_/*S*_FO_, of iron oxide nanospheres of diameter *d* by a layer of Y silicate of thickness *t*_YS_ from eq 2 (see Section 1.5 in SI).

$$\frac{S_{\mathrm{YS}}}{S_{\mathrm{FO}}}=\frac{d}{6t_{\mathrm{YS}}}\cdot \frac{\rho_{\mathrm{YS}}M_{\mathrm{YS}}}{\rho_{\mathrm{FO}}M_{\mathrm{YS}}}\frac{x}{\left(1-x\right)}$$ 2

where ρ and *M* are the densities and molar mass of the considered oxides, respectively, and *x* is the relative Y concentration.

For sample Y10 (*x* = 0.1), we observe a sharp increase in average aspect ratio (Figure 1e). We can also see that for Y10, the thickness of the Y silicate layer around the nanorods is very thin, ∼2 nm, but it completely coats them (see SI, Figure S21), suggesting that at *x* = 0.1, it is possible to achieve the critical coverage required to trigger the anisotropic growth of nanorods. Indeed, taking *d* = 25 nm (maximum d of ε-Fe_2_O_3_ prepared by the bulk sol–gel method without Y), and *t*_YS_ = 2 nm, eq 2 gives a relative coverage of less than 20% for *x* = 0.05, which increases to 40% for *x* = 0.1. Thus, for this Y concentration, it seems plausible that an effective coverage of a certain width all around the perimeter parallel to [100] can be achieved, decreasing the diffusion across the interfaces covered by Y_2_Si_2_O_7_. This creates the conditions for the nanocrystal to grow along [100], with more Y silicate from dissolving nanoparticles filling unoccupied sites at the specific facets for which it has an affinity, pushing the three-phase contact line of SiO_2_–Y_2_Si_2_O_7_–Fe_2_O_3_ toward the rod apex, thereby further contributing to the anisotropic growth, as outlined in Figure 3e.

We performed atomistic simulations to elucidate the underlying mechanism of this process and, more specifically, the absence of Y silicate on the surfaces perpendicular to [100]. We performed geometry relaxations (see details in Section 1.4 of SI) based on the fast and accurate MACE-MP machine learning potential,^39^ trained with the high-quality DFT data of the Materials Project,^40^ considering starting structures from the same database for ε-Fe_2_O_3_ (mp-542896) and Y_2_Si_2_O_7_ (mp-561531). We obtained structure optimizations and calculations of the interaction energies, Δ*E*_int_, when different crystal faces of ε-Fe_2_O_3_ are put in contact with Y_2_Si_2_O_7_. The simulation results are collected in Table 1, and Figure 4 presents illustrative snapshots of the interfaces. Our calculations indicate that Y_2_Si_2_O_7_ has a different affinity for the different faces of ε-Fe_2_O_3_, being the most energetically favorable case the interaction with the [001] surface and the least favorable case the interaction with the [100] surface. The order of magnitude of the surface energies reported in Table 1 is similar to surface energy differences reported for the growth of nanorods.^41^ The geometry optimizations (Figure 4, right column) show that the Y atoms tend to coordinate with the oxygen atoms exposed in ε-Fe_2_O_3_ surfaces. Since each ε-Fe_2_O_3_ surface has a different availability of oxygen atoms, as illustrated in the left column of Figure 4, our calculations predict that Y silicate will preferentially cap the surfaces with a larger density of oxygen atoms, providing an atomistic interpretation to the experimental observations.

**Table 1:** Interaction Energies of Y_2_Si_2_O_7_ and ε-Fe_2_O_3_ ^a^

ε-Fe_2_O_3_ surface	[100]	[010]	[001]	[00–1]
Δ*E*_int_ (eV)	–200.6	–223.49	–231.2	–203.2
Δ*E*_int_/*A* (eV/nm^2^)	–131.65	–146.64	–151.71	–133.32

a Results obtained for the interaction energy Δ*E*_int_ due to the addition of a slab of Y_2_Si_2_O_7_ over different faces of ε-Fe_2_O_3_ from geometry optimization calculations (see eq S2 in SI, Section 1.4). We also give the results normalized to the contact area (*A* = 1.524 nm^2^) between the Y_2_Si_2_O_7_ and the ε-Fe_2_O_3_ substrate.

**Figure 4:**
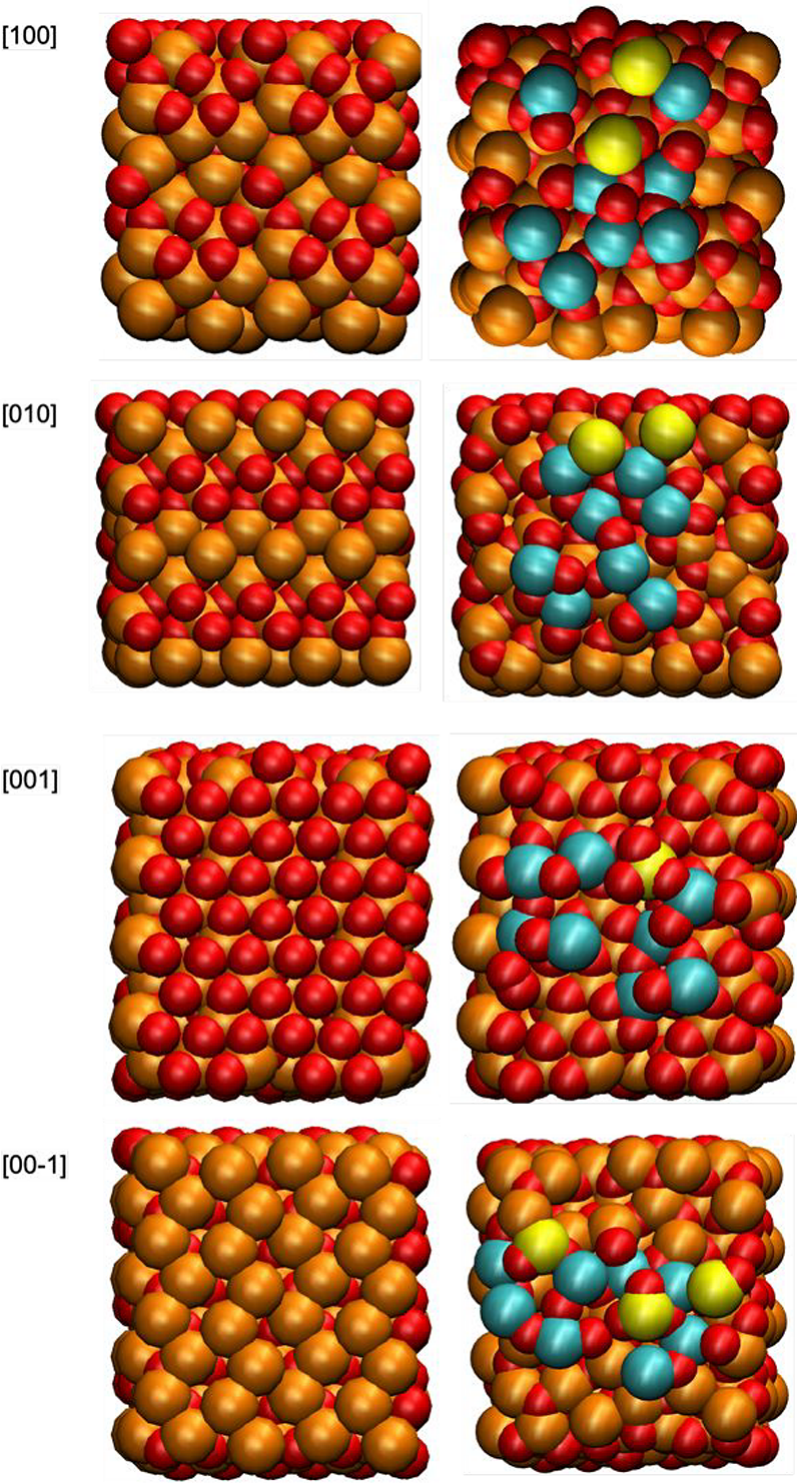
Snapshots of the ε-Fe_2_O_3_ crystal faces considered in the calculations before and after contact with a Y_2_Si_2_O_7_ slab (optimized structures). In order to facilitate visualization, we only show the atoms of the Y_2_Si_2_O_7_ slab in contact with the ε-Fe_2_O_3_ surface. The atoms are shown as van der Waals spheres: Fe (orange), O (red), Y (cyan), and Si (yellow). Image made with VMD.^42^

Our findings suggest that rare-earth silicate capping layers can act as high-temperature surfactants. This is a new element to expand the toolbox to control metal oxide nanostructures at high temperatures, which comprises strategies such as the use solid-phase oxygen buffers, recently illustrated in the iron oxide-silica nanostructures.^43^ In fact, the suitability of rare-earth silicates as high-temperature coatings is already being exploited in application areas not related to nanomaterials and magnetism. Thanks to their high thermal stability, chemical inertness and good adhesion are applied as environmental barrier coatings to enhance the durability of engineering materials such as SiC ceramic matrix composites in harsh operation conditions.^44^ This versatility and compatibility with different types of materials point to the possibility of using rare-earth silicates for controlling the growth of a broader palette of nanomaterials at high temperatures.

### c-Application to ε-(Fe_1–*x*_Cr_*x*_)_2_O_3_ Nanoparticles with the Enhanced Anisotropy Field

Taking a first step in this direction, we have applied the new method to the synthesis of ε-(Fe_1–*x*_Cr_*x*_)_2_O_3_ nanoparticles, which was crucial in unraveling that Cr^3+^ substitutions increase the magnetic anisotropy of ε-Fe_2_O_3_. In prior studies on Cr^3+^ substitutions in ε-Fe_2_O_3_, we and others had reported that this cation induces a decrease in magnetic anisotropy.^45,46^ We recently noticed that extending the Cr^3+^substitution beyond 10 at % resulted in a strong reduction in the ε-(Fe_1–*x*_Cr_*x*_)_2_O_3_ nanoparticle sizes prepared by the bulk sol–gel method. The blue curve of Figure 5a shows that the average crystal size obtained from synchrotron XRD for 15 at % Cr^3+^ is just one-half of that of ε-Fe_2_O_3_. Nickel et al. have recently signaled and discussed this marked impact of Cr^3+^ on particle size, proposing that it can arise either because the substitution increases the bulk formation energy or because it decreases the surface energy.^46^ We prepared another series of ε-(Fe_1–*x*_Cr_*x*_)_2_O_3_ nanoparticles with 0, 7.5, and 15 at % Cr^3+^ by the new Y-assisted bulk sol–gel synthesis, keeping the same temperature and annealing time as in reference^45^ (see Section 1, SI). The average crystal size of these new ε-(Fe_1–*x*_Cr_*x*_)_2_O_3_ nanoparticles (green curve in Figure 5a) varied between 40 and 47 nm, showing almost no dependence on the Cr content. Between the two possibilities considered by Nickel et al., our findings support the hypothesis of a decrease of interfacial energy driven by the Cr^3+^ substitution, which due to the nanoparticle growth by Ostwald ripening will result in smaller sizes according to eq 1. Indeed, the marked size reduction is not observed for the nanoparticles prepared with the new approach, suggesting that the Y silicate capping layer that has been discussed above plays the role of a surfactant that increases the interfacial energy with the surrounding silica matrix.

**Figure 5:**
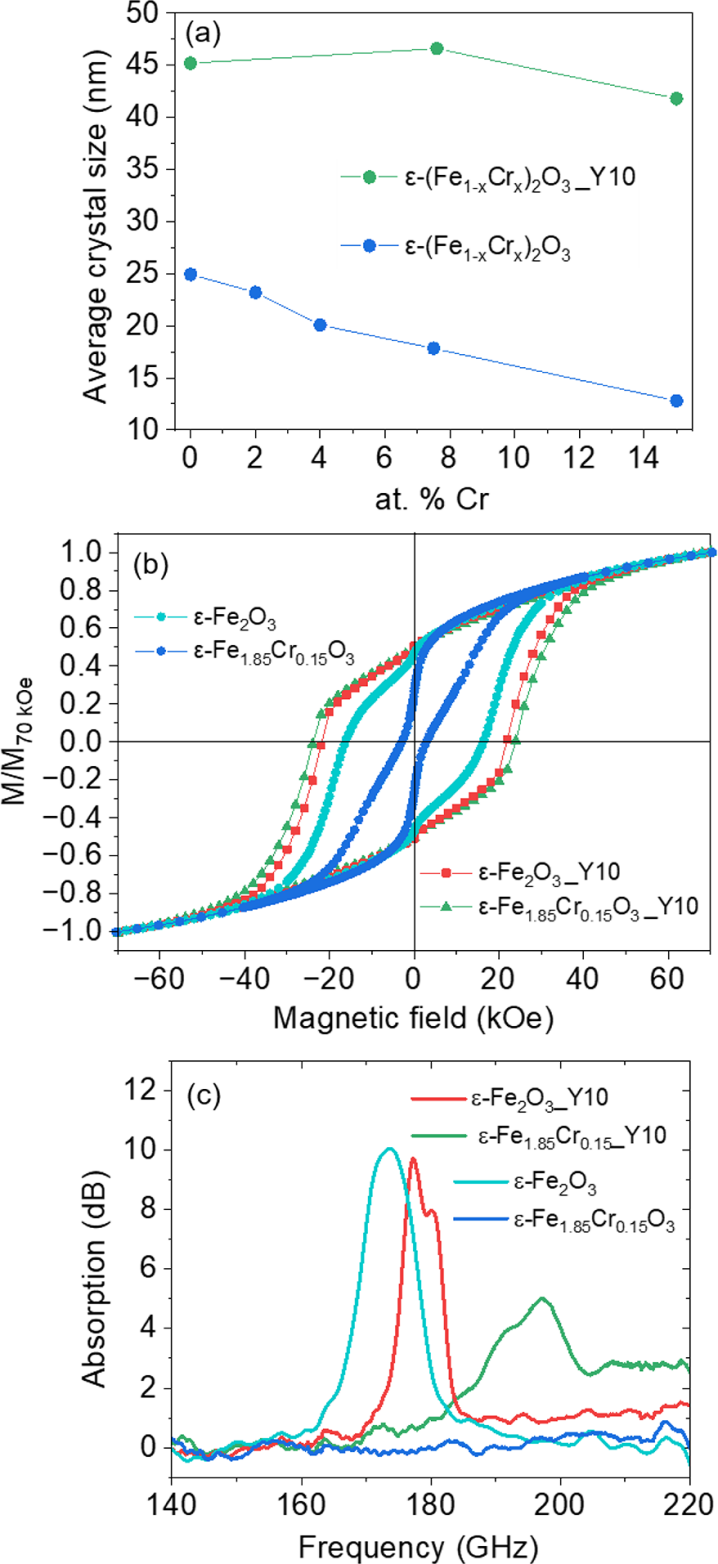
(a) Evolution of the average crystal sizes obtained from the Rietveld refinement of ε-(Fe_1–*x*_Cr_*x*_)_2_O_3_ nanoparticles, prepared with Y^3+^ (Y10, *x* = 0.1) and without any additive, as a function of molar Cr^3+^ content; (b) magnetic hysteresis loops (*M*/*M*_70 kOe_ vs *H*) of ε-Fe_2_O_3_ and ε-Fe_1.85_Cr_0.15_O_3_ (i.e., for a substitution of 7.5 at % Cr^3+^, *x* = 0.075) prepared, respectively, in the absence of any additives and in the presence of Y^3+^ (Y10); (c) quasi-optical measurements of the ferromagnetic resonance for ε-Fe_2_O_3_ and ε-Fe_1.85_Cr_0.15_O_3_ nanoparticles prepared with Y^3+^ (Y10) and without any additives.

Figure 5b presents room temperature hysteresis loops for ε-Fe_1.85_Cr_0.15_O_3_ nanoparticles of different average sizes: 47 nm for the sample prepared with the addition of Y^3+^ and 18 nm for the one synthesized without Y^3+^. The Figure evidences the strong impact of particle size on the magnetic anisotropy, with an 8-fold increase in the coercive field from 2.9 kOe of the small particles to about 24 kOe for the ones prepared with the new synthesis method. For comparison, the figure also shows the measurements for ε-Fe_2_O_3_ rods (Y10 sample discussed above) and nanoparticles prepared by the standard bulk sol–gel method (eFO sample discussed above), which present lower coercive fields, indicating that the substitution is increasing the magnetic anisotropy. This is confirmed by measurements of the natural ferromagnetic resonances (FMR) of these materials in a quasi-optical transmissometer between 140 and 220 GHz (Figure 5c). The FMR absorption corresponding to ε-Fe_2_O_3_ rods (sample ε-Fe_2_O_3__Y10) is sharper than for ε-Fe_2_O_3_ prepared without Y, it is slightly shifted to higher frequencies and presents two appreciable modes. For ε-Fe_1.85_Cr_0.15_O_3_ rods prepared with Y^3+^, the FMR is at a significantly higher frequency and also split into two modes. The two FMR modes in the two nanorod samples have been related to a splay of the orientation of magnetic moments at the rod edges, which arises from the competition between exchange and demagnetizing energies.^47^ In contrast, the FMR of ε-Fe_1.85_Cr_0.15_O_3_ nanoparticles prepared without Y^3+^ was not detected in the studied range, as one could expect from its low coercive field values. The FMR frequency for ε-Fe_1.85_Cr_0.15_O_3__Y10 is comparable to that reported for Rh-doped ε-Fe_2_O_3_ for equivalent substitution level,^16^ but with a more cost-compatible element, just like recently observed for Mn substitutions.^48^ In all these cases, the substitution of Fe^3+^ is accompanied by a decrease in magnetization (see the decrease of the high-field magnetization with Cr^3+^ substitution in S22), which also results in a lower absorption, clearly observed for ε-Fe_1.85_Cr_0.15_O_3_ in Figure 5c. Since the FMR frequency is proportional to the anisotropy field *H*_a_ = *K*/μ_0_ *M*_s_, in these cases the increase of FMR frequencies seems to be associated with the progressive diminution of the saturation magnetization *M*_s_ upon metal substitution rather than related to a significant increase of the magnetic anisotropy.

## Conclusions

This work describes a modification of the bulk sol–gel method for preparing ε-Fe_2_O_3_ in large amounts that allows for increasing the size and aspect ratio of the crystals. In these larger ε-Fe_2_O_3_ nanorods, the remanent magnetization does not show any trace of the undesired superparamagnetic relaxation, contrary to the nanoparticle systems obtained by the standard bulk sol–gel approach. The new method is based on the addition of Y^3+^ or other trivalent rare earths such as La^3+^ and Dy^3+^, which are too large to substitute Fe^3+^ in the ε-Fe_2_O_3_ structure. With the presence of Y^3+^, the dense glassy matrix with ε-Fe_2_O_3_ crystals obtained by heating the silica gels undergoes a binodal decomposition. The secondary glassy phase, of composition ∼Y_2_Si_2_O_7_, coats ε-Fe_2_O_3_ nanoparticles, preferentially capping surfaces with a large density of exposed oxygen atoms while avoiding certain surfaces, such as the [100], where this density is low. From an atomistic point of view, this behavior can be explained by the strong affinity of Y^3+^ for oxygen, an interpretation that is supported by theoretical calculations employing machine-learning force fields.

Another key finding of this study is the existence of a dual Ostwald ripening mechanism that governs the growth of ε-Fe_2_O_3_ nanorods and allows understanding their size increase with the presence of trivalent rare earths. The RE^3+^ silicate capping layer selectively formed around the rod axes increases the surface energy and/or mass transfer coefficients, compared to that of the rod apexes, free of RE silicate. The contrasting ripening dynamics of the surfaces with and without RE silicate promote the preferential growth of surfaces perpendicular to the [100] crystallographic direction. Additionally, the larger aspect ratios obtained by using La^3+^ instead of Y^3+^ suggest an inverse correlation between aspect ratio and cationic field strength.

For practical purposes, the role of the RE silicate capping is in many respects analogous to that of a surfactant in solution chemistry and could be used to modify interfacial energies and control the microstructure of materials and composites at high temperatures. Our findings highlight the potential of phase-separated glassy media, where one of the glasses can act as a surface structuring agent on crystals growing by Ostwald ripening. This opens new possibilities for the high-temperature synthesis of nanostructures with controlled morphologies. These prospects broaden the view of the glassy phase as a supercooled liquid to encompass phenomena analogous to those exploited by colloid chemistry in solutions.

## Supplementary Material



## Notes

Experimental methods and methodologies on the synthesis of nanoparticles, determination of size distributions, structural characterization, atomistic simulations, calculation of surface coverage, and measurements of magnetic and ferromagnetic resonance response (Figures S1, S2 and Tables S1, S2); magnetic relaxation dependence on particle size (Figure S3); X-ray diffraction patterns of ε-Fe_2_O_3_ prepared with Y^3+^(Figure S4 and Table S3); TEM images, size and volume distributions of ε-Fe_2_O_3_ prepared with Y^3+^ for compositions not presented in the manuscript (Figure S5); STEM and TEM characterization of the etched Y15 sample prepared by bulk sol–gel synthesis with Y^3+^ (Figures S6–S11 and Tables S4, S5); STEM and XRD characterization of the phase-separated glass of the same Y:Si content as Y10 but without Fe (Figure S12); TEM and STEM characterization and size distributions of samples prepared by bulk sol–gel synthesis with Y^3+^, La^3+^, and Dy^3+^ (Figures S13–S15); TEM images and size distributions of ε-Fe_2_O_3_ prepared with Bi^3+^, Cd^3+^, and Sb^3+^ for the composition *x* = 3 (Figure S16); X-ray diffraction patterns of ε-Fe_2_O_3_ prepared with Y^3+^, Bi^3+^, Cd^3+^, and Sb^3+^ for the composition *x* = 3 (Figure S17 and Table S6); evolution of nanoparticle sizes with annealing time (Figures S18 and S19); evolution of nanoparticle sizes with temperature for Y10 quenched samples (Figures S20); STEM characterization of the etched Y10 sample prepared by bulk sol–gel synthesis with Y^3+^ (Figure S21); and magnetization of ε-(Fe_1–*x*_Cr_*x*_)_2_O_3_ nanoparticles prepared with Y^3+^ (Figure S22) (PDF)
