# Supplementary material for: Rare-Earth Silicates as High-Temperature Surfactants for the Controlled Synthesis of ε‑Fe2O3 Nanoparticles
Source: J Am Chem Soc. 2025 Sep 4;147(37):33403–12. doi: 10.1021/jacs.5c05058 (PMC12447499; doi:10.1021/jacs.5c05058)
Supplement: Supplementary file 1 [file ja5c05058_si_001.pdf]

Supporting Information

Rare earth silicates as high-temperature surfactants for the controlled synthesis of ε-Fe_2O_3 nanoparticles

Naureen Khanam, Zheng Ma, Sergi Ortiz Ropero, Nico Dix, Ana Vila Costa, Judit Oró-Solé, José Luis García-Muñoz, Jordi Faraudo, Martí Gich*

Institut de Ciència de Materials de Barcelona, ICMAB-CSIC, carrer dels Til·lers, 08193 Cerdanyola del Vallès (Barcelona) Spain

Section 1. Experimental methods and methodologies

1. Synthesis of ε-Fe_2O_3 nanoparticles

Bulk sol-gel synthesis of ε-Fe_2O_3:

$Fe(NO_3)_3{\cdot}9H_2O$ from *Thermo Scientific* and tetraethyl orthosilicate (TEOS) from *Sigma Aldrich* were used as precursors of the metal oxide and SiO_2, respectively. Milli-Q water and absolute ethanol (*PanReac AppliChem*) were used as solvents. 20 mL TEOS was used as the basis for the calculation, keeping the molar ratio [Fe^{3+}]: [Si^{4+}] = 0.25. The molar ratio of TEOS: ethanol: H_2O was fixed at 1:5:6. First, stoichiometric amounts of H_2O and ethanol were mixed in a beaker, then the desired stoichiometric amount of $Fe(NO_3)_3{\cdot}9H_2O$ was dissolved. After the metal precursor was dissolved, TEOS was added to this solution at 0.7 ml/min using a syringe pump at a high stirring rate (~700 rpm) to ensure homogenous mixing. After adding TEOS, the solution was left under stirring for 2 hours and equally distributed into four Petri dishes of 8.5 cm diameter that were kept inside a plastic box for further gelation and syneresis. The gelation took place after 4 to 7 days. The as-prepared gels were ground manually and then was subject to an annealing process in air at different temperatures (950 ºC, 1000 ºC, 1050 ºC and 1100 ºC) for 3 hours at 80 ℃/h heating rate, followed by natural cooling inside the furnace. Finally, the nanoparticles were collected by etching the as-prepared powders in a 10 M NaOH solution at 80 ℃ under reflux for 48 hours. The different samples were labelled as BSG_T, were T is the temperature of annealing.

Reverse micelle sol-gel synthesis of ε-Fe_2O_3 with Ba^{2+}:

The chemicals used in this synthesis were $Fe(NO_3)_3{\cdot}9H_2O$ and NH_4OH from *Thermo Scientific*; n-octane, tetraethyl orthosilicate (TEOS), and cetyltrimethyl ammonium bromide(CTAB) from *Sigma Aldrich*; $Ba(NO_3)_2$ from *Alfa Aesar* and 1-butanol from *MEDICHEM Diagnostica*. Highly purified Milli-Q water was used where necessary. Two microemulsions (RM-A and RM-B) were prepared in two separate beakers. The molar contents of the precursors are presented in Table S1. For the oil phase of the microemulsions, stoichiometric amounts of n-octane, 1-butanol, and CTAB were added into two beakers and stirred thoroughly to have a homogenous mixture.

Table S1 Concentration of precursors in the synthesis of ε-Fe_2O_3 by reverse micelle sol-gel method

Sample	CTAB mmol	1-butanol mmol	n-octane mmol	H_2O mmol	$Fe(NO_3)_3{\cdot}9H_2O$ mmol	$Ba(NO_3)_2$ mmol	NH_3 mmol	TEOS mmol
RMSG_Ba_0.05_1000	9.7	39	110	330	0.74	0.037	30	6.7
RMSG_Ba_0.1_1000	9.7	39	110	330	0.74	0.074	30	6.7

The aqueous phase of the RM-A was prepared in another beaker by mixing stoichiometric amounts of H_2O, $Ba(NO_3)_2$, and $Fe(NO_3)_3{\cdot}9H_2O$ with continuous stirring. The aqueous phase of RM-B was prepared separately with a stoichiometric amount of NH_4OH (28-30%) and H_2O. Both aqueous phases were added to the respective beakers containing the oil and surfactant mixtures drop by drop. After stirring for 30 minutes, RM-B was added drop by drop to RM-A at a high stirring rate. Finally, TEOS was added to the solution and stirred for 20 hours. The supernatant was separated by centrifuging at 5000 rpm for 10 minutes. Subsequently, the precipitate was washed first by a 1:1 mixture of chloroform and methanol, and then by pure methanol. Finally, the precipitate was heated at 80 ℃/h to 1000 ℃ in air and held at that temperature for 4 hours. Then, the sample was left to cool down inside the furnace. The silica matrix was etched using a 3 M NaOH solution at 80 ℃ under reflux for 24 hours.

Bulk sol-gel synthesis of ε-Fe_2O_3 with Ba^{2+} by impregnation

The preparation method for this sample follows the one used to prepare sample BSG_1100 up to the addition of the TEOS step. After adding the TEOS, the solution was poured into a plastic beaker instead of a Petri dish to have enough gel height for the impregnation. After gelation, the xerogel was transferred to a beaker containing 100 mL of 6 mM $Ba(NO_3)_2$ solution. After 20 hours of soaking, the solution was removed using a Pasteur pipette, and the gel was dried at 60 °C for 24 hours, followed by grinding and subsequent annealing at 1100 °C in air for 3 hours using an 80 °C/h heating ramp and an eventual natural cooling process. Finally, the as-prepared powders were etched with a 10 M NaOH solution at 80 °C under reflux for 48 hours.

Bulk sol-gel synthesis of ε-Fe_2O_3 with Y nitrate:

ε-Fe_2O_3 nanoparticles stabilized with Y were prepared with slight modifications to the same protocol used for the bulk sol-gel synthesis of ε-Fe_2O_3 as described above. The Y^{3+} precursor was yttrium(III) nitrate hexahydrate ($Y(NO_3)_3.6H_2O$) from *Thermo Scientific*, which is easily soluble in the hydroethanolic medium. A series of seven samples with varying [Y^{3+}]: [Fe^{3+}] molar ratios were prepared. Sample specifications with the corresponding molar ratios are given in Table S2. 10 mL TEOS for samples Y1, Y3, Y5, and Y10 and 20 mL TEOS for samples Y15, Y20, and Y25 were taken as the basis of all calculations. The molar ratio of TEOS: ethanol: H_2O was fixed for all samples to 1:5:6. The only modification in the synthesis was to add the stoichiometric amount of $Y(NO_3)_3{\cdot}6H_2O$ in the hydroethanolic medium before adding the iron precursor to ensure the salt's dissolution. The as-prepared ground gels were annealed at 1100 °C for 3 hours in air using a heating rate of 80 °C /h.

Table S2: Synthesis compositions of ε-Fe_2O_3 prepared with Y additions in the sol

Sample name	x= [Y]: [Y+Fe]	[Fe]: [Si]	[Y]: [Si]	[Fe+Y]:[Si]
Y1	0.01	0.248	0.002	0.25
Y3	0.03	0.240	0.007	0.25
Y5	0.05	0.234	0.012	0.25
Y10	0.10	0.218	0.025	0.24
Y15	0.15	0.201	0.035	0.24
Y20	0.20	0.185	0.047	0.23
Y25	0.25	0.171	0.057	0.23

Bulk sol-gel synthesis of ε-Fe_2O_3 with other rare earth (Dy, La Ce) nitrates:

For other rare earth additives, a similar protocol to that of the bulk sol-gel synthesis with Y^{3+} was used. To evaluate the effect of La as an additive, $La(NO_3)_3{\cdot}6H_2O$ from *Alfa Aesar* was used as a precursor. Two different La content compositions were evaluated, with a molar ratio *x*= $[La^{3+}]$:$[La^{3+}+Fe^{3+}]$= 0.03 and 0.1, and the corresponding samples were labeled as La3 and La10, respectively.

In the case of Dy, $Dy(NO_3)_3{\cdot}5H_2O$ from *Sigma Aldrich* was used as a precursor. Two different Dy content compositions were evaluated, with a molar ratio *x*= $[Dy^{3+}]$:$[Dy^{3+}+Fe^{3+}]$= 0.03 and 0.1, and the corresponding samples were labeled as Dy3 and Dy10, respectively.

In the case of Ce, a series of samples with a varying molar ratio, x=$[Ce^{3+}]$: $[Ce^{3+}+Fe^{3+}]$= 0.03, 0.05, 0.1, 0.2, and 0.25, were prepared using Ce(NO3)3·6H2O from Alfa Aesar as the Ce^{3+} precursor. The corresponding samples were labeled as Ce3, Ce5, Ce10, Ce20, and Ce25, respectively.

To evaluate the effects of Bi^{3+}, Cd^{2+} and Sb^{3+}, we used $Bi(CH_3COO)_3$, $Cd(CH_3COO)_2{\cdot}2H_2O$ and $SbCl_3$ from *Sigma Aldrich*, following a protocol analogous to that of the synthesis with Y^{3+}. In the case of $Bi^{3+}$and Sb^{3+}, glacial acetic acid from *Labbox Labware S.L.* was used as a chelating agent for stabilization of the metal cations.

Preparation of materials to study the influence of annealing time at 1100ºC on growth

A series of Y10 gels were annealed at 1100 °C for different times ranging from 0 h (quenching upon reaching 1100ºC) to 18 h, as follows. The whole batch of prepared gel was heated to 1100ºC and quenched. Then, fractions of this initial batch were introduced into the furnace held at 1100ºC to be annealed for different times and quenched again. Similar procedures were adopted for gels prepared with La^{3+} and without rare earth cations.

2. Particle size and volume distributions

The size distributions of the particles (width, length and aspect ratios) were obtained from the analysis of low-resolution TEM images using the ImageJ software [1], by sampling more than 150 particles. For the volume distributions, we took into account that particles of different shapes (spherical and nano-rods) can be found within a given sample. The volume was approximated to that of a sphere for particles with aspect ratios below 1.05. The particles with larger aspect ratios (nanorods) were considered as cylinders with parabolic caps at their apexes, and their volumes were computed from the length *L*, width of the rod cylindrical part *w*, and cap height *h* (see Equation S1 and Figure S1a below), with the particularity that we experimentally established, sampling a large number of flat-lying particles which appeared in a symmetric projection, that *w/h*~2.9 with a standard deviation of 0.3 regardless of the length or aspect ratio *L/w* (Figure 1b,c below).

$$V_{nanorod} = V_{cylinder} + 2 \cdot V_{paraboloid} = \left[\pi\left(\frac{w}{2}\right)^2 \cdot (L-2h)\right] + 2 \cdot \left[\frac{\pi}{2}\left(\frac{w}{2}\right)^2 \cdot h\right] \approx$$

$$\left[\pi\left(\frac{w}{2}\right)^2 \cdot (L-2 \cdot w/2.9)\right] + 2 \cdot \left[\frac{\pi}{2}\left(\frac{w}{2}\right)^2 \cdot w/2.9\right] \qquad \text{(Equation S1)}$$

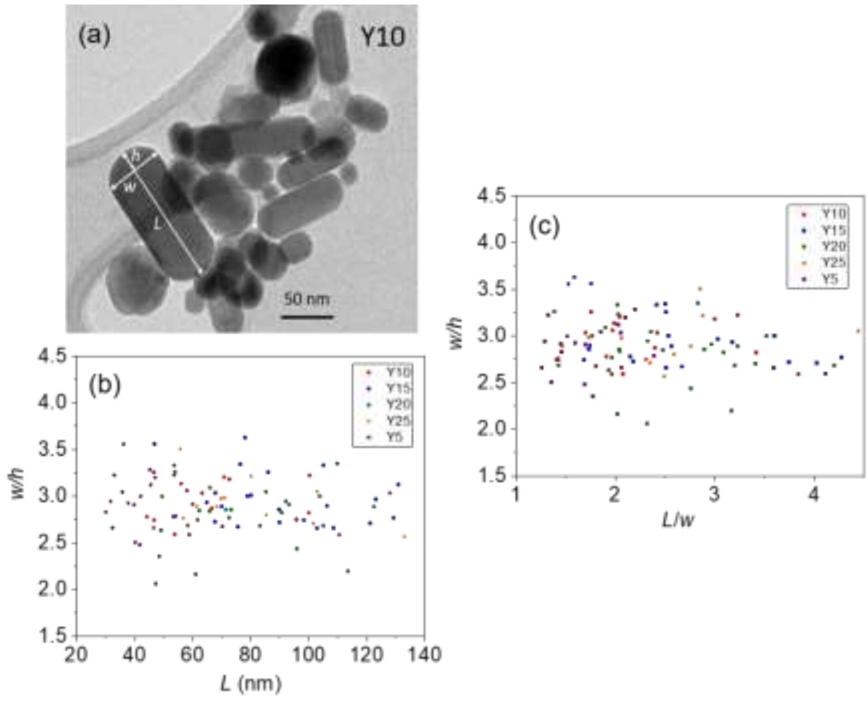

Figure S1: TEM image illustrating the dimensions measured to compute the nanorod volumes (a). It is found that for all the samples *w/h*~2.9 ±0.3 irrespective of the Y content, nanorod length (b) and aspect ratio (c).

The sampled volumes approximately followed log-normal distributions. To compare the particle sizes as a function of Y content, we calculated average values of nanorod lengths, widths, aspect ratios and volumes, to which we associated the 95 % confidence interval as an error bar.

3. Crystallographic and structural characterizations using XRD, SEM and TEM techniques

XRD measurements were performed at the ICMAB X-ray diffraction service using the following equipment: A Bruker D8 Advance A25 diffractometer with Debye-Scherrer geometry using Mo Kα radiation (λ=0.7107 Å); a Siemens D-5000 diffractometer a Bragg-Brentano geometry with Cu Kα radiation (λ=1.5406 Å).

Synchrotron X-ray powder diffraction was performed at the BM04-MSPD beamline of the ALBA Synchrotron Light facility (Barcelona, Spain) using a MYTHEN detector. A 0.5-mm-diameter borosilicate glass capillary was loaded with the sample and kept spinning during data acquisition, which was performed using a wavelength λ= 0.4428 Å. The value of λ was calibrated using a silicon standard. Rietveld refinement of XRD patterns was done using the MAUD software [2].

Transmission electron microscopy (TEM) low-resolution images and diffraction patterns were acquired with a JEOL JEM-1210 electron microscope operating at 120 kV with an ORIUS 831SC600 Gatan camera. High-resolution images were acquired with a ThermoFisher Scientific Spectra 300 monochromatic Scanning-Transmission Electron Microscope operating at 300 kV, equipped with a Super-X EDX (Energy Dispersive X-Ray) spectrometer of the JEMCA facility at the ALBA Synchrotron, Spain. The EDX spectra were analysed with the Velox software (version 3.14.0 ThermoFisher Scientific).

4. Computer Simulations

Simulation Methods

We have performed structure optimizations and energy calculations of different crystal faces of ε-Fe_2O_3 in contact with $Y_2Si_2O_7$, starting from the structures available at the Materials Project [3] database (structures mp-542896 and mp-561531, respectively). The level of theory employed in the calculations of the energy and forces between atoms is the MACE-MP machine learning (ML) potential, specifically the MACE-MP-0 medium version, model 20231203-mace-128-L1_epoch-199 [4], which is a fast and accurate ML potential covering 86 chemical elements that has been trained over high quality DFT data covering relaxation trajectories of 1.6 M bulk crystals from the Materials Project database. The atomic coordinates were relaxed using the Broyden–Fletcher–Goldfarb–Shanno (BFGS) optimization method as implemented in the Atomistic Simulation Environment (ASE) python library [5] using MACE-MP as calculator in ASE. The force tolerance for the BFGS calculation was 0.05 eV/Å.

Simulation Protocol

First, we used ASE to generate a 4x2x2 supercell of ε-Fe_2O_3 from the unit cell of the Materials Project mp-542896 structure (40 atoms and 5.215 Å × 8.952 Å × 9.596 Å dimensions). The obtained supercell has 640 atoms and measures 20.860 Å × 17.904 Å

$\times$ 19.192 Å. We also generated a slab of $Y_2Si_2O_7$ with dimensions 10.230 Å $\times$ 16.280 Å $\times$ 14.898 Å and 176 atoms generated as a 2x2x2 replica of the unit cell of the mp-561531 structure (22 atoms, dimensions 5.115 Å x 8.140 Å x 7.449 Å).

In order to build a simplified model mimicking the interaction of Y atoms from $Y_2Si_2O_7$ with the surfaces of ε-Fe_2O_3 observed in the experiments, we have created new structures by putting into close contact the [0-10] face of the $Y_2Si_2O_7$ slab (which exposes 8 Y atoms and no Si atoms) with different faces of the ε-Fe_2O_3 structure generated as described above. Snapshots (ball and stick representations) of the ε-Fe_2O_3 crystal considered in the calculations showing the crystal faces considered in the calculations. An image made with VMD [6], can be seen below in Figure S2. The atom coordinates of the resulting structure ($Y_2Si_2O_7$ slab over different surfaces of ε-Fe_2O_3) were relaxed using the BFGS optimization method as described above. The energy associated with the interaction (ΔE_{int}) is computed from the energy of the relaxed composite system (E_{rel}) and the energies of the ε-Fe_2O_3 and $Y_2Si_2O_7$ structures:

$$\Delta E_{int} = E_{rel} - E(\varepsilon\text{-}Fe_2O_3) - E(Y_2Si_2O_7). \qquad \text{(Equation S2)}$$

The energy is then normalized to the contact area between the $Y_2Si_2O_7$ slab and ε-Fe_2O_3.

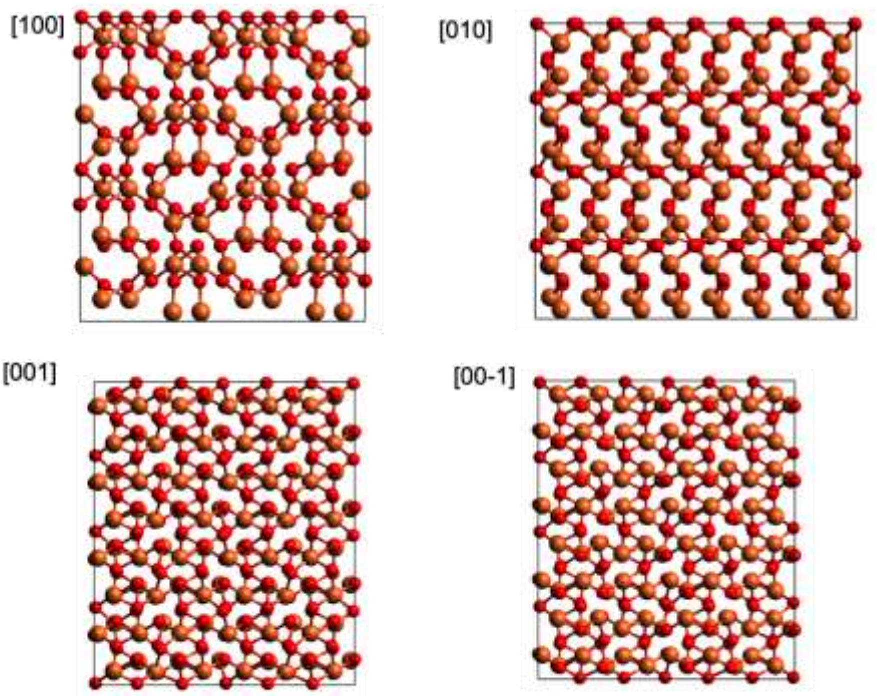

Figure S2: Snapshots (ball and stick representations) of the ε-Fe_2O_3 crystal considered in the calculations showing the crystal faces considered in the calculations. Image made with VMD [6].

5. Estimating surface coverage of Fe_2O_3 nanoparticles by Y silicate

One can estimate the fraction of Fe_2O_3 nanoparticles surface covered by Yttrium silicate as a function of x from the ratio of the total surfaces of $Y_2Si_2O_7$ and Fe_2O_3, S_{YS}/S_{FO}. For this purpose, we can consider on the one hand that for low x, Fe_2O_3 nanoparticles are spherical and that S_{FO} can be expressed as the total volume of Fe_2O_3 times the surface to volume ratio $S_{FO}=(6/d)\cdot V_{FO}$., where d is the nanoparticle diameter. On the other hand, we can express S_{YS} as the total volume of $Y_2Si_2O_7$ divided by the characteristic thickness of the Yttrium silicate layer on nanoparticles, t_{YS}.

$$\frac{S_{YS}}{S_{FO}} = \frac{d}{6t_{YS}} \cdot \frac{V_{YS}}{V_{FO}} = \frac{d}{6t_{YS}} \cdot \frac{\rho_{YS} m_{YS}}{\rho_{FO} m_{YS}} = \frac{d}{6t_{YS}} \cdot \frac{\rho_{YS} M_{YS}}{\rho_{FO} M_{FO}} \frac{x}{(1-x)} \qquad \text{(Equation S3)}$$

Where ρ_i, m_i and M_i are the density, total mass and molar mass of the oxides, and x is the relative concentration of Y with respect to iron, x=[Y]/([Y]+[Fe]).

Taking ρ_{FO}= 5 g/cm^3, ρ_{YS}~4 g/cm^3, M_{FO}=159.69 g/mole, M_{YS}=345.98 g/mole, d=25 nm and t_{YS}= 2 nm, we can estimate that for x=0.05, the relative coverage is less than 20 %, while for x=0.1 it increases to 40%. From these calculations, we can understand that the growth of nanorods is triggered for x=0.1, considering that with a nanoparticle coverage by Y silicate above 40 %, an effective coverage of a certain width all around the perimeter parallel to [100] is possible, decreasing the diffusion across the interfaces covered by $Y_2Si_2O_7$, which will start to grow.

6. Magnetometry measurements

Time-dependent magnetic measurements were carried out using the vibrating sample magnetometry (VSM) option of a physical properties measurement system (PPMS) from Quantum Design system. The measurements were carried out for the nanoparticles embedded inside the silica matrix in order to avoid any movement of the magnetic nanoparticles in such long measurements. The nanoparticles embedded in the silica matrix were mixed with high-temperature Zircar alumina cement adhesive and put inside a gelatine capsule before mounting on the VSM heater stick. A 90 kOe magnetic field was applied to saturate the samples before applying zero field, and the time-dependent magnetization measurements were performed for 10^4 seconds at 300 K.

Room-temperature magnetic hysteresis loops were collected for etched ε-Fe_2O_3 and ε-$(Fe_{1-x}Cr_x)_2O_3$ prepared in the presence and absence of Y additives using a SQUID magnetometer from Quantum Design Inc. First, we mounted a known weight of the powder in a gelatine capsule, and then a certain amount of cotton fibers was put inside the capsule to prevent the movement of the powder during data acquisition. The capsule was then inserted inside a transparent drinking straw. Finally, after ensuring the easy centering of the specimen, the straw was mounted to the SQUID platform. A 70 kOe magnetic field was applied to saturate the samples before applying an opposite field of -70 kOe and again applied up to 70 kOe to obtain the magnetic moment for the applied

field. The magnetization at each applied field was normalized to the magnetization at 70 kOe.

7. Ferromagnetic resonance (FMR) spectroscopy

The millimetre wave measurements were performed with a VNA (Keysight: N5227B) in a quasi-optical (QO) system (Thomas Keating Ltd.) [7] in the frequency range of 140-220 GHz (WR5 band, using WR5.1-VNAX Vector Network Analyzer Extender by Virginia Diodes, Inc.). The QO setup is comprised by frequency multipliers, horn antennas, and focusing mirrors. Mirrors and antennas were arranged in the focusing mode, where the Gaussian beam emitted by the horn antennas was focused on the sample. The beam size at the sample holder position is approximately 7.5 mm in diameter. The powders of ε-Fe_2O_3 nanoparticles were pressed into a cylindrical Teflon container with a diameter of 10 mm (inner diameter 9 mm) and a thickness of 4 mm, and then placed in the sample holder for mm-wave measurement. The VNA was calibrated with Thru-Reflect-Line (TRL) calibration, then transmission parameters were measured, where the absorption in [dB] is $A = -10log\frac{|S_{21}|^2}{(1-|S_{11}|^2)}$, with the measured scatter parameters: being S_{21} the transmission coefficient and S_{11} the reflection coefficient.

Section 2. Magnetic relaxation dependence on particle size for ε-Fe_2O_3

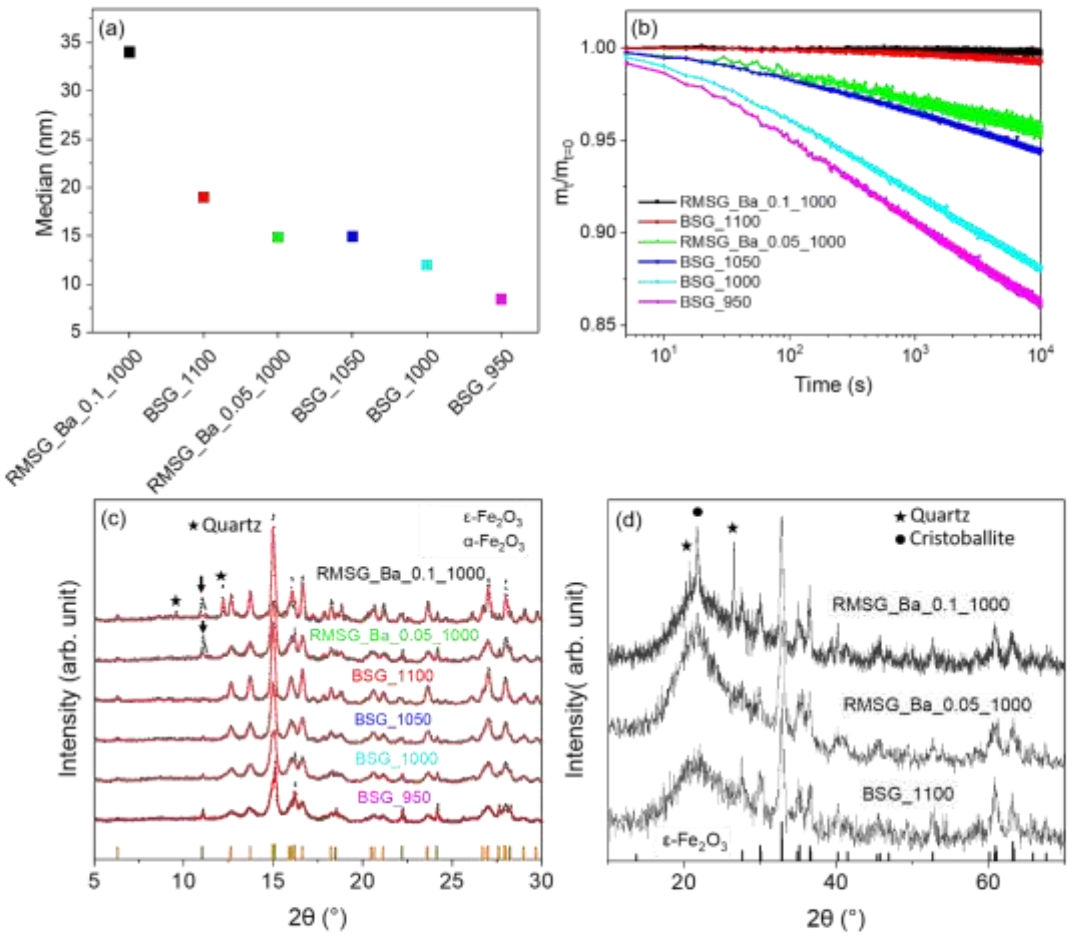

Figure S3: (a) Average diameter of the samples prepared by different methods and annealed for 3 h at different temperatures. RMSG and BSG refer to reverse micelle sol-gel and bulk sol-gel, respectively; **(b)** Time evolution of the remanent magnetization, expressed as the remanent magnetic moment normalized to the initial remanent moment, for the same samples of panel **(a)**. **(c)** X-ray diffraction pattern for etched samples prepared by reverse micelle sol-gel method and sol-gel methods. Red lines correspond to the Rietveld refinement; **(d)** Diffraction patterns for non-etched samples prepared by reverse micelle sol-gel method and sol-gel method (annealing temperature 1100 ℃).

Section 3. X-ray diffraction of ε-Fe_2O_3 prepared by bulk sol-gel synthesis with Y^{3+}

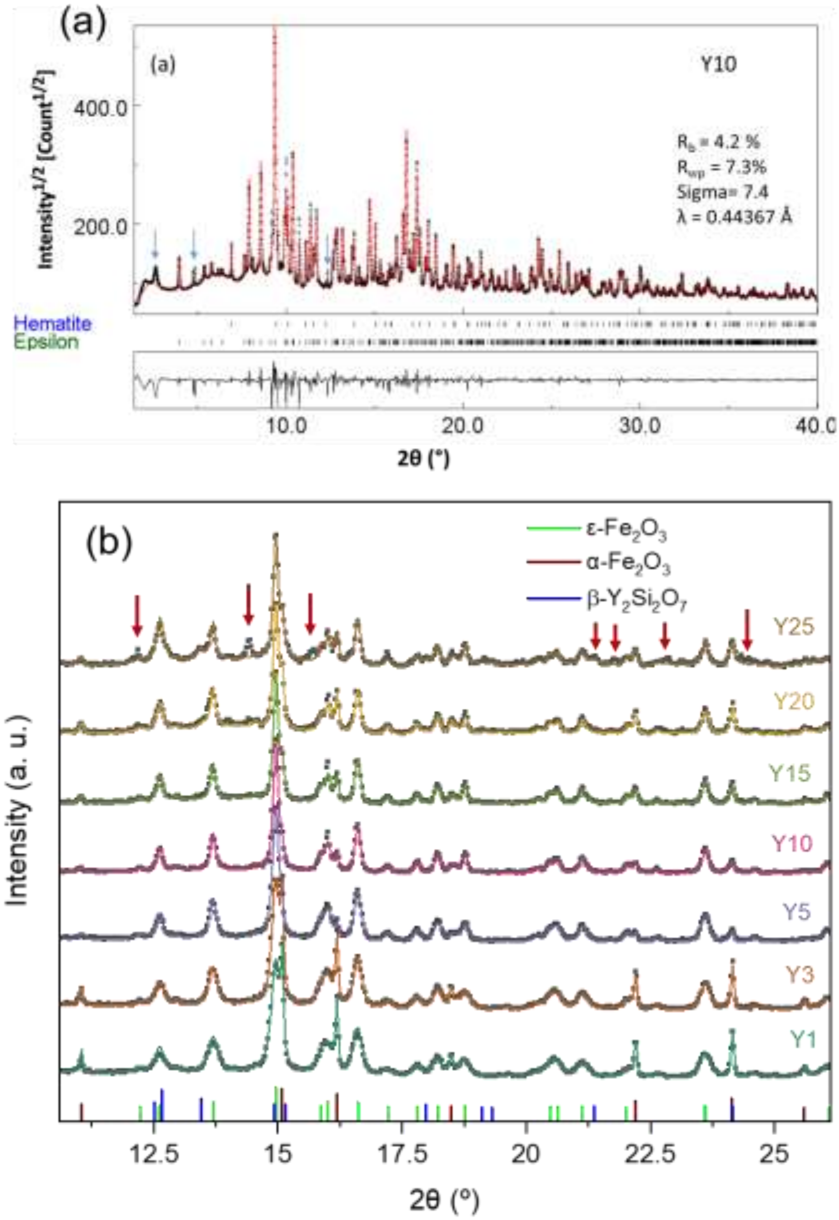

Figure S4. **(a)** The Synchrotron X-ray powder diffraction pattern of etched sample Y10 (*x*= 0.1); **(b)**The comparison of etched samples Y10, Y20, and Y25 shows the emergence of β-$Y_2Si_2O_7$ and another unknown phase. The vertical blue arrows in the synchrotron XRD pattern of sample Y10 presented in panel **(a)** could not be identified and were not observed in the pattern previously recorded in a diffractometer (bottom pattern in panel **(b)**).

Table S3. Lattice parameters and Goodness of Fit factors collected from Rietveld refinement of the Y-added etched samples

Sample name	Y1	Y3	Y5	Y10	Y15	Y20	Y25
a (Å)	5.0937(5)	5.0922(4)	5.0930(3)	5.0926(3)	5.0938(3)	5.0921(3)	5.0931(6)
b (Å)	8.7894(9)	8.7881(8)	8.7874(4)	8.7857(5)	8.7845(6)	8.7854(7)	8.787(1)
c (Å)	9.4700(8)	9.4721(7)	9.4732(3)	9.4727(5)	9.4736(6)	9.4732(7)	9.475(1)
Cell Colume (Å^3)	423.9(1)	423.8(6)	423.96(3)	423.82(4)	423.9(1)	423.7(1)	424.0(1)
Crystal size (nm)	26.2 ± 0.1	31.7 ± 0.3	39.6 ± 0.3	52.8 ± 0.1	57 ± 1	54 ± 1	54 ± 1
wt. % α-Fe_2O_3	15	16	5.5	9	15	17	13
wt. % $Y_2Si_2O_7$	0	0	0	0	0	7	28
R_b (%)	4.8	4.6	4.5	4.7	4.4	4.3	5.5
R_{wp} (%)	6.3	5.8	5.7	5.9	5.5	5.4	7.5
Sigma	1.3	1.2	1.2	1.3	1.3	1.3	1.7

Section 4. TEM images, particle size and volume distributions of ε-Fe_2O_3 prepared by bulk sol-gel synthesis with Y^{3+}

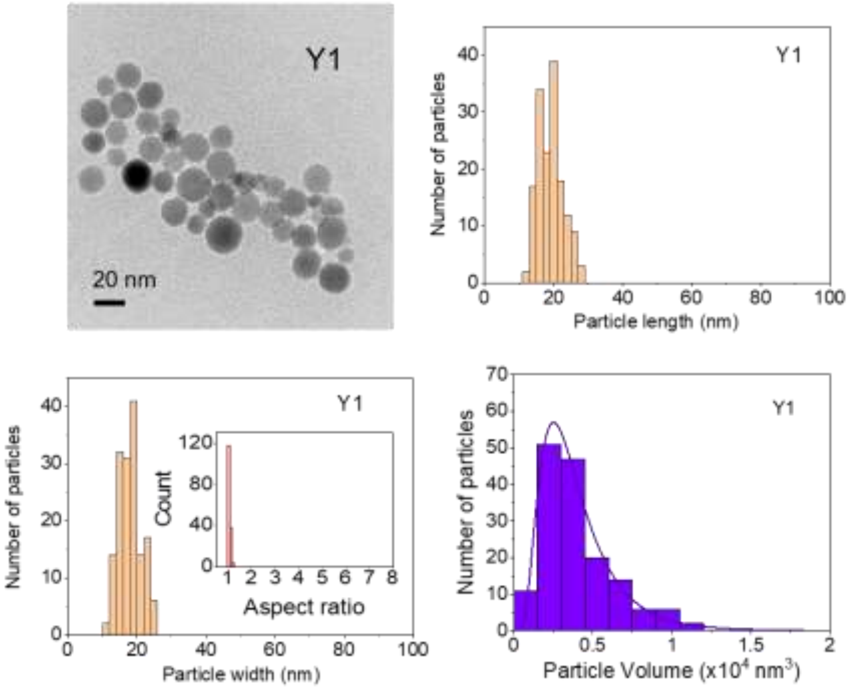

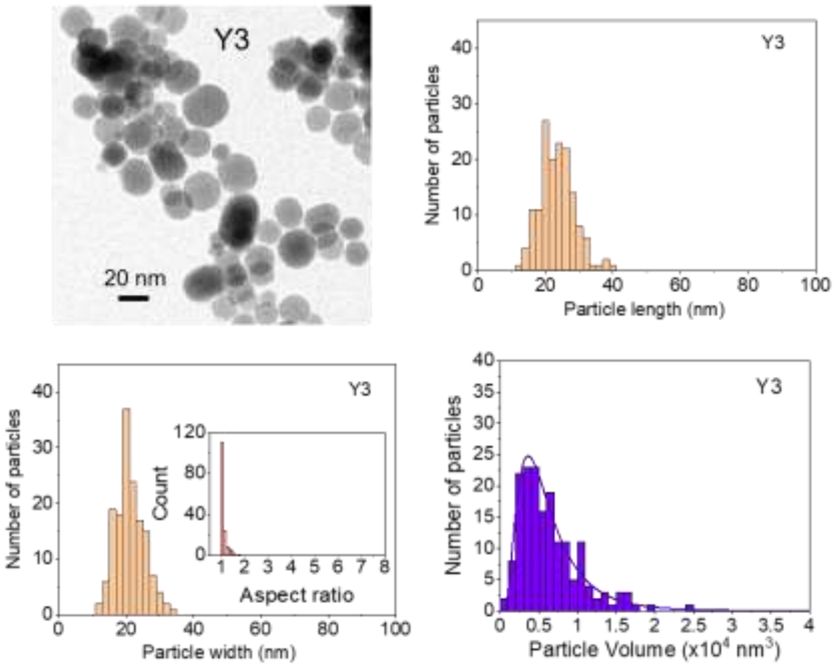

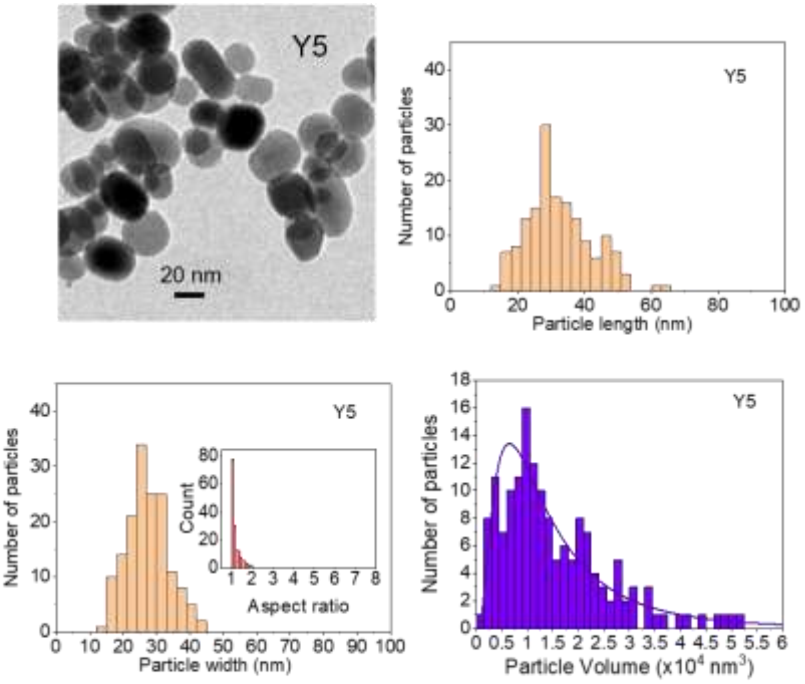
Y5
20 nm
Y5
Number of particles
Particle length (nm)
Y5
Number of particles
Particle width (nm)
Count
Aspect ratio
Y5
Number of particles
Particle Volume (x10^4 nm^3)

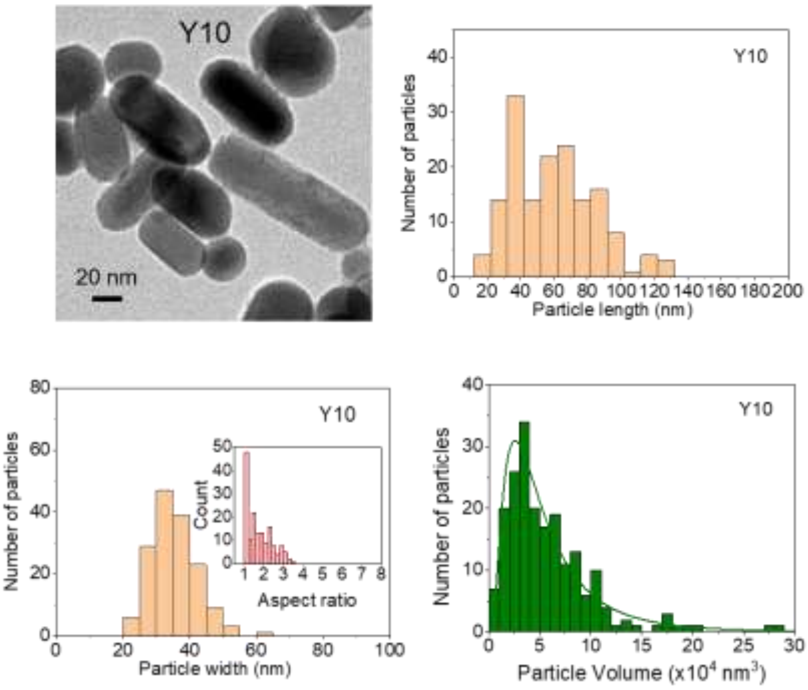
Y10
20 nm
Y10
Number of particles
Particle length (nm)
Y10
Number of particles
Particle width (nm)
Count
Aspect ratio
Y10
Number of particles
Particle Volume (x10^4 nm^3)

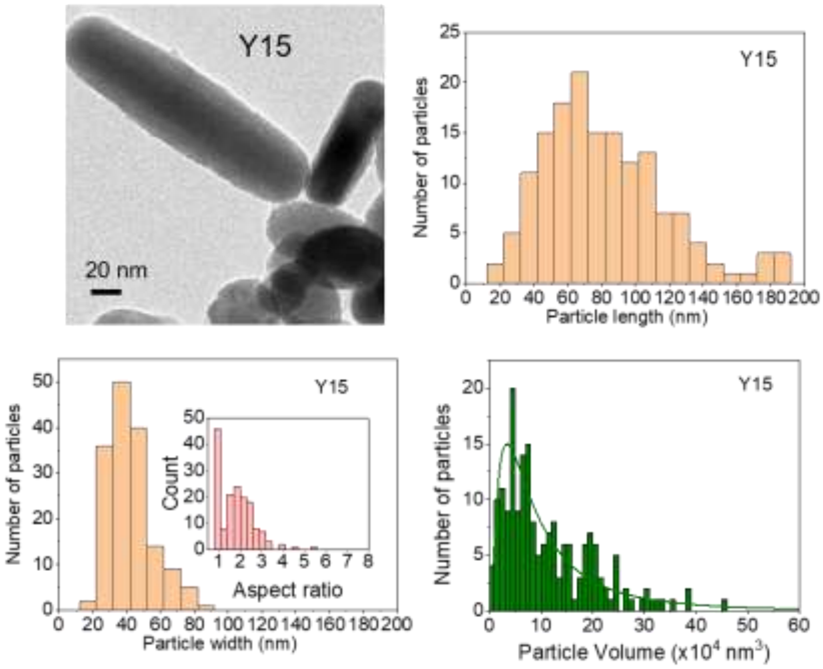
Y15
20 nm
Number of particles
Particle length (nm)
Particle width (nm)
Count
Aspect ratio
Particle Volume (x10^4 nm^3)

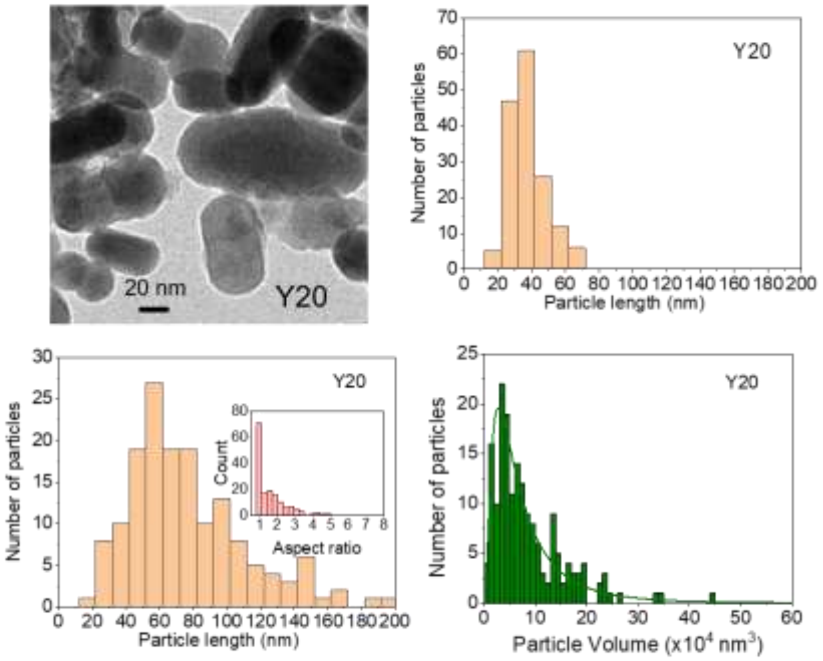
Y20
20 nm
Number of particles
Particle length (nm)
Count
Aspect ratio
Particle Volume (x10^4 nm^3)

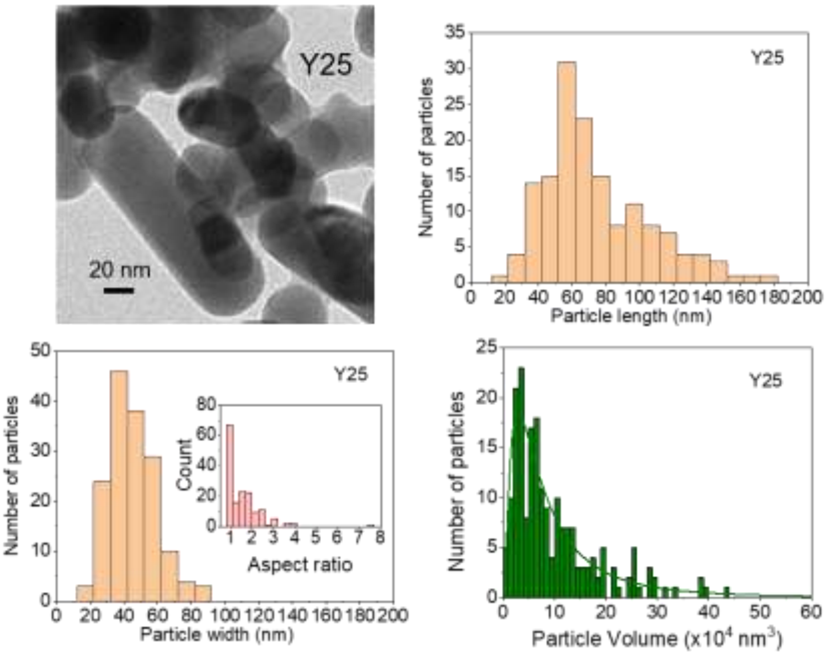

Figure S5. TEM images of ε-Fe_2O_3 nanoparticles and their particle size, aspect ratios and volume distributions prepared with different Y^{3+} contents: *x*= 0.01 (Y1), *x*= 0.03 (Y3), *x*= 0.05 (Y5), *x*= 0.1 (Y10), *x*= 0.15 (Y15), *x*= 0.2 (Y20) and *x*= 0.25 (Y25).

Section 5. STEM characterization of etched Y15 sample prepared by bulk sol-gel synthesis with Y^{3+}

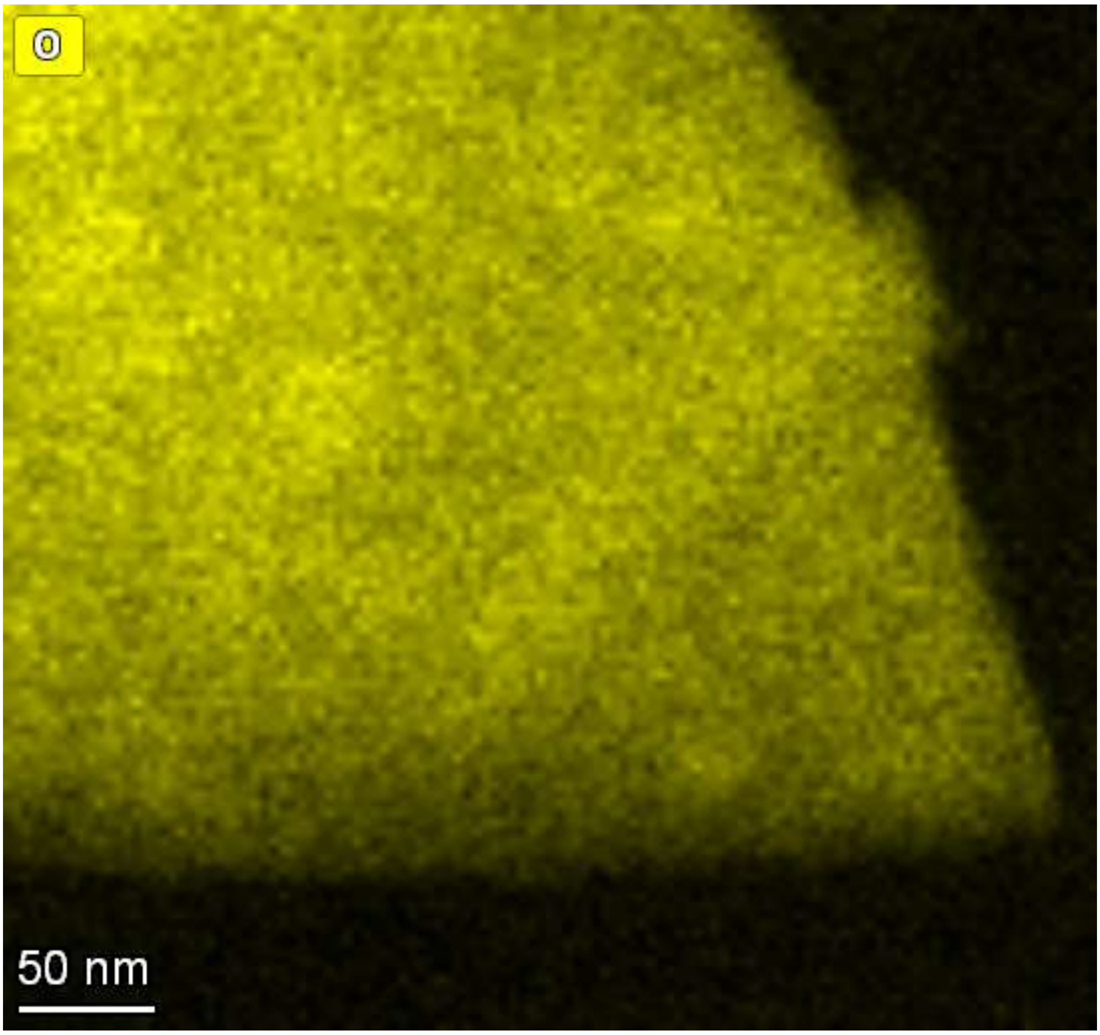

Figure S6. EDX Oxygen elemental map of sample Y15 corresponding to the same zone as in Figure 2(a) of the main manuscript.

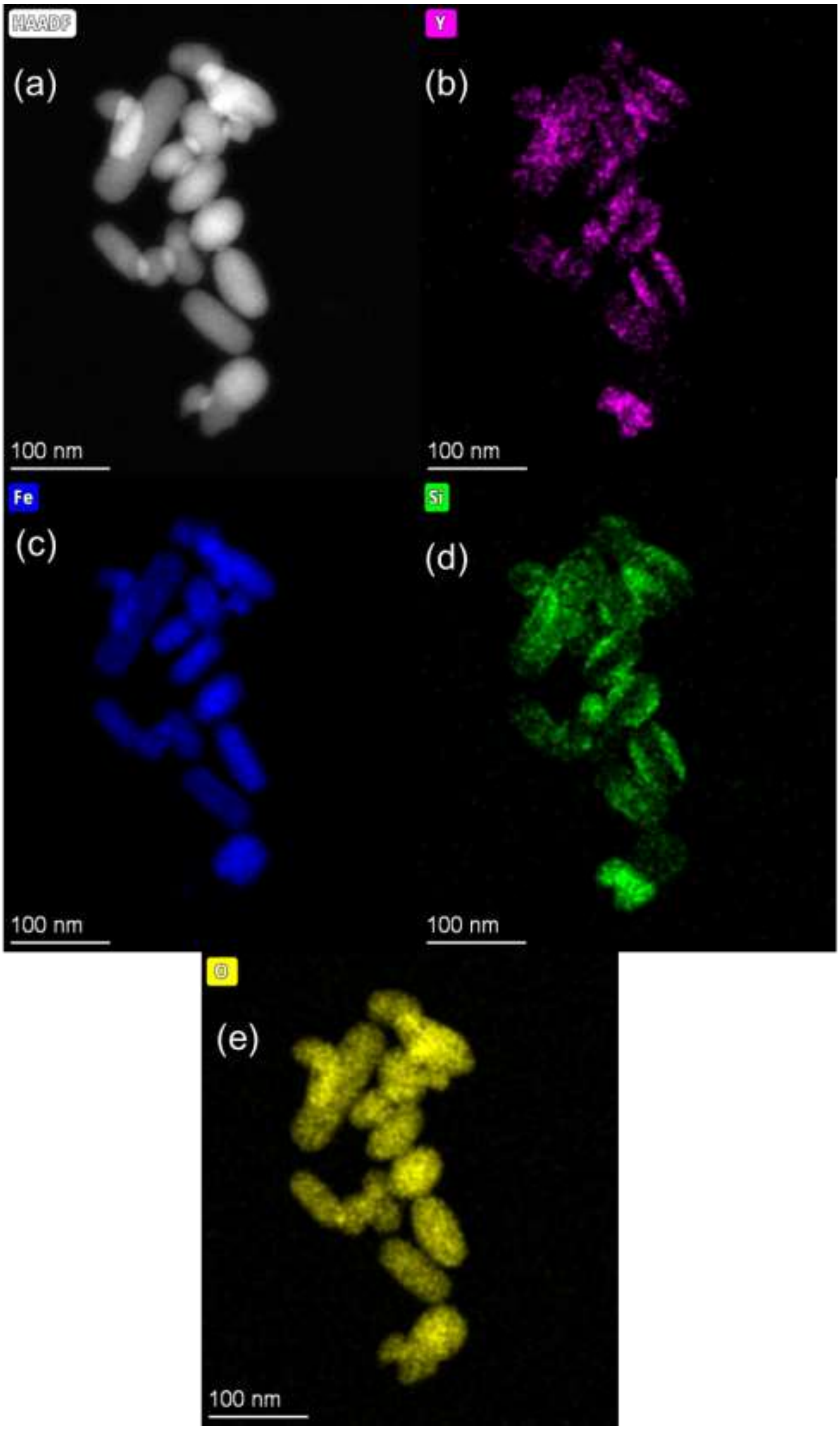

Figure S7. (a) HAADF image of a group of nanorods of sample Y15 after etching out the silica matrix; **(b)** to **(e)** are elemental distribution maps of the same zone of the panel **(a)** obtained by EDX, respectively corresponding to Y, Fe, Si, and O.

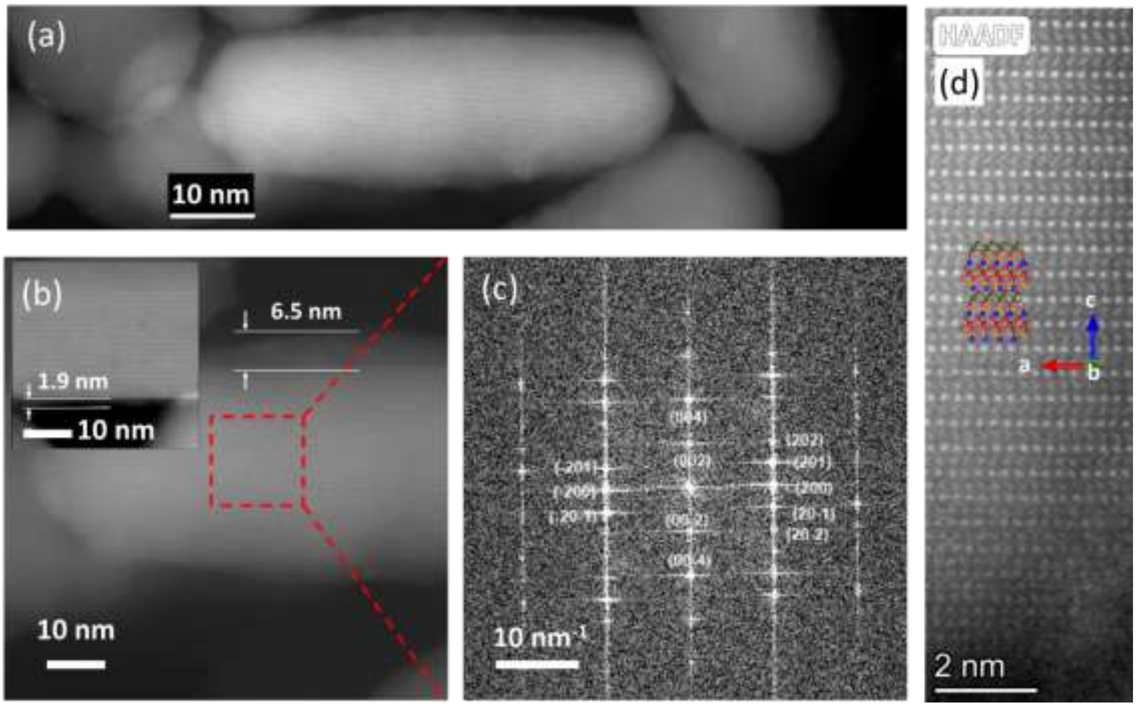

Figure S8. (a) HAADF image of a single nanorod prepared with Y concentration *x*= 0.15 corresponding to a view along the [010] zone axis; **(b)** A closer view of the same nanorod and in the inset a single nanorod prepared with Y concentration *x*=0.1; **(c)** FFT of the dotted red square of **(b)**; **(d)** overlap of the ε-Fe_2O_3 lattice structure on the HAADF image.

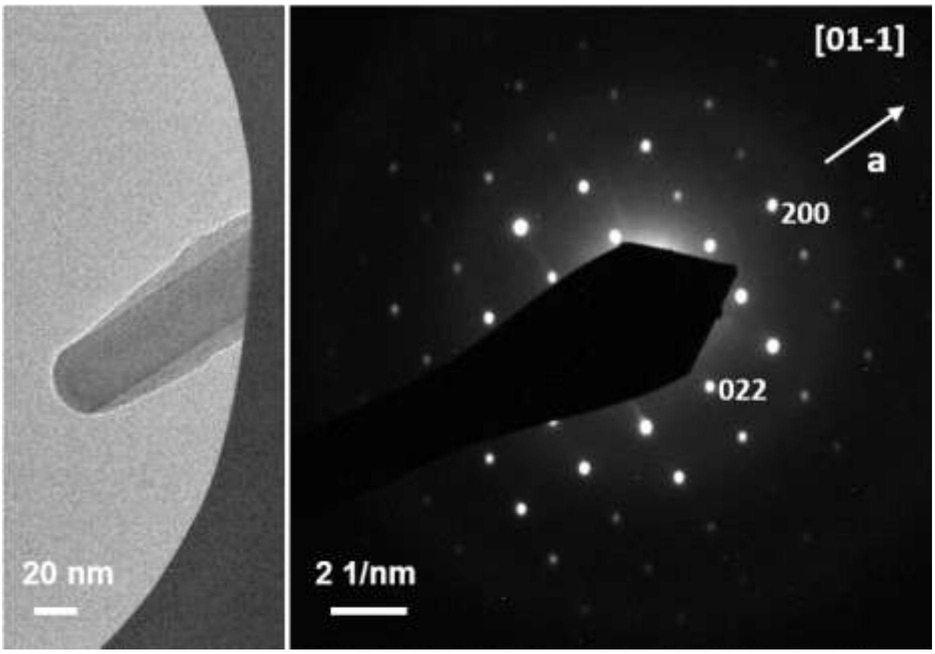

Figure S9. TEM images of an individual particle of the Y15 sample. **(a)** Bright-field TEM image with selected area electron diffraction (SAED) aperture. **(b)** Corresponding SAED pattern, indexed to the [01-1] zone axis of ε-Fe_2O_3. The absence of additional diffraction features suggests that the particle coating is amorphous.

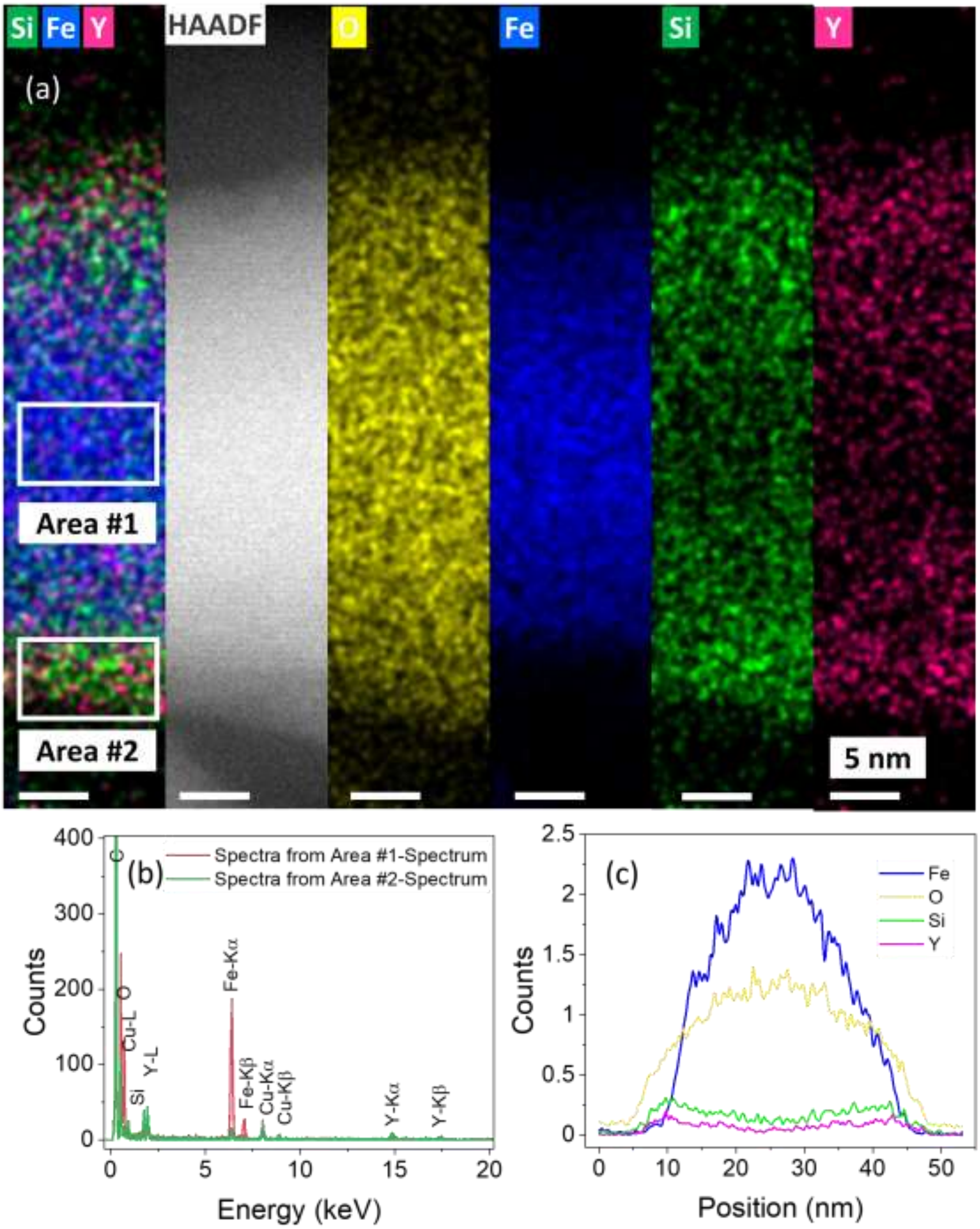

Figure S10. (a) Elemental maps and HAADF of a single nanorod section; **(b)** EDS spectra from Area #1 and Area #2 in (a); (c) Fe, O, Si and Y concentration profiles along a direction perpendicular to the rod axis.

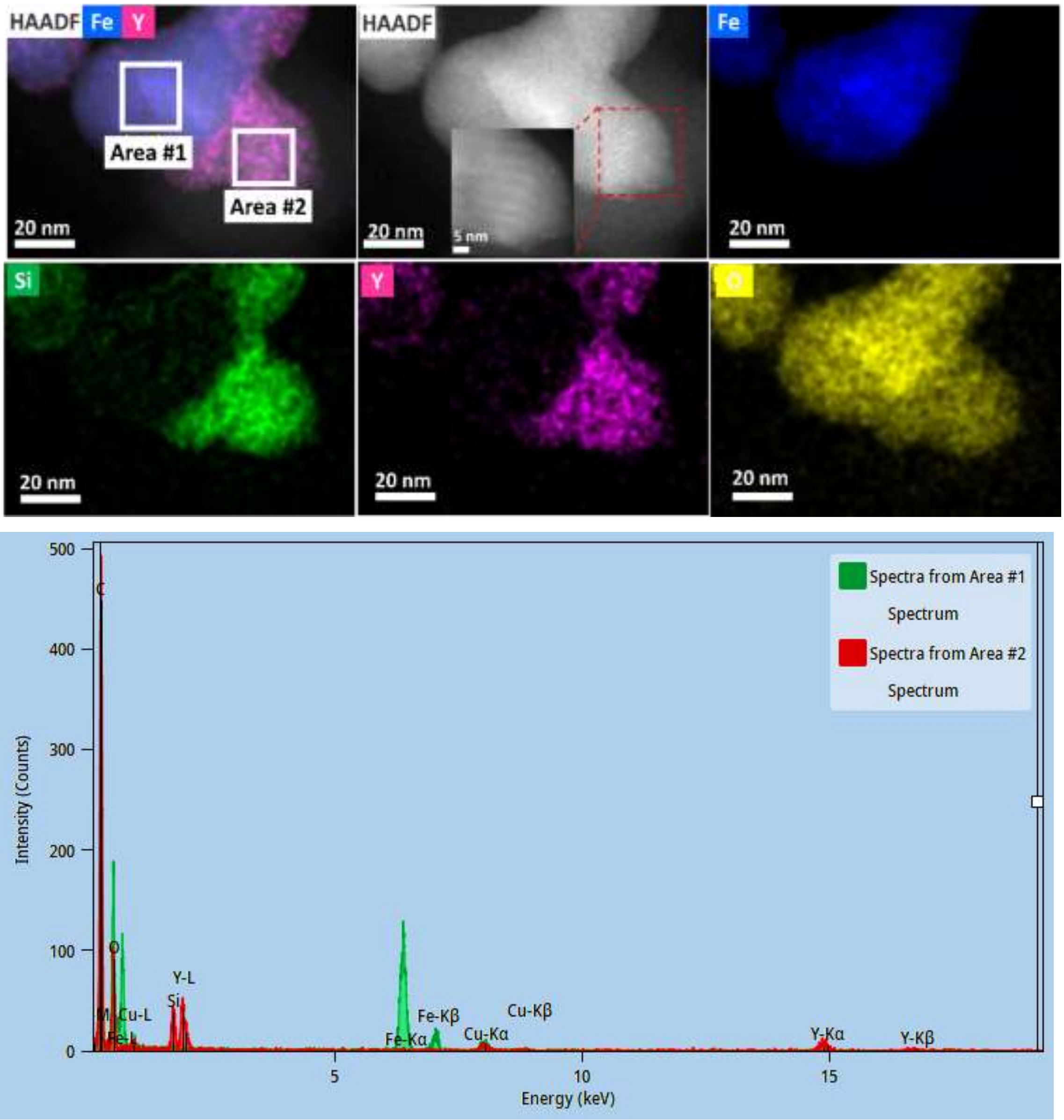

Figure S11. (Top) EDX elemental maps and HAADF image of a ε-Fe_2O_3 nanoparticle in contact with a zone rich in Y and Si. The closer view of the zone rich in Y and Si (inset of HAADF image) shows that it is crystalline; **(Bottom)** The spectra collected for the two areas indicated in the top image.

Table S4: Analysis of EDX spectrum from Area #1

Z	Element	Family	Atomic Fraction (%)	Atomic Error (%)	Mass Fraction (%)	Mass Error (%)	Fit Error (%)
8	O	K	64.70	2.98	34.44	2.94	0.63
14	Si	K	0.42	0.11	0.39	0.11	17.09
26	Fe	K	34.54	3.01	64.17	3.06	0.29
39	Y	L	0.34	0.06	1.00	0.18	10.16

Table S5: Analysis of EDX spectrum from Area #2

Z	Element	Family	Atomic Fraction (%)	Atomic Error (%)	Mass Fraction (%)	Mass Error (%)	Fit Error (%)
8	O	K	64.02	2.58	32.42	2.29	0.59
14	Si	K	17.22	2.78	15.31	2.69	1.75
26	Fe	K	0.50	0.10	0.89	0.18	13.68
39	Y	L	18.26	1.96	51.38	3.47	0.57

Section 6. Characterization of the silica glass with the same content of Y^{3+} as sample Y10 but without Fe^{3+}.

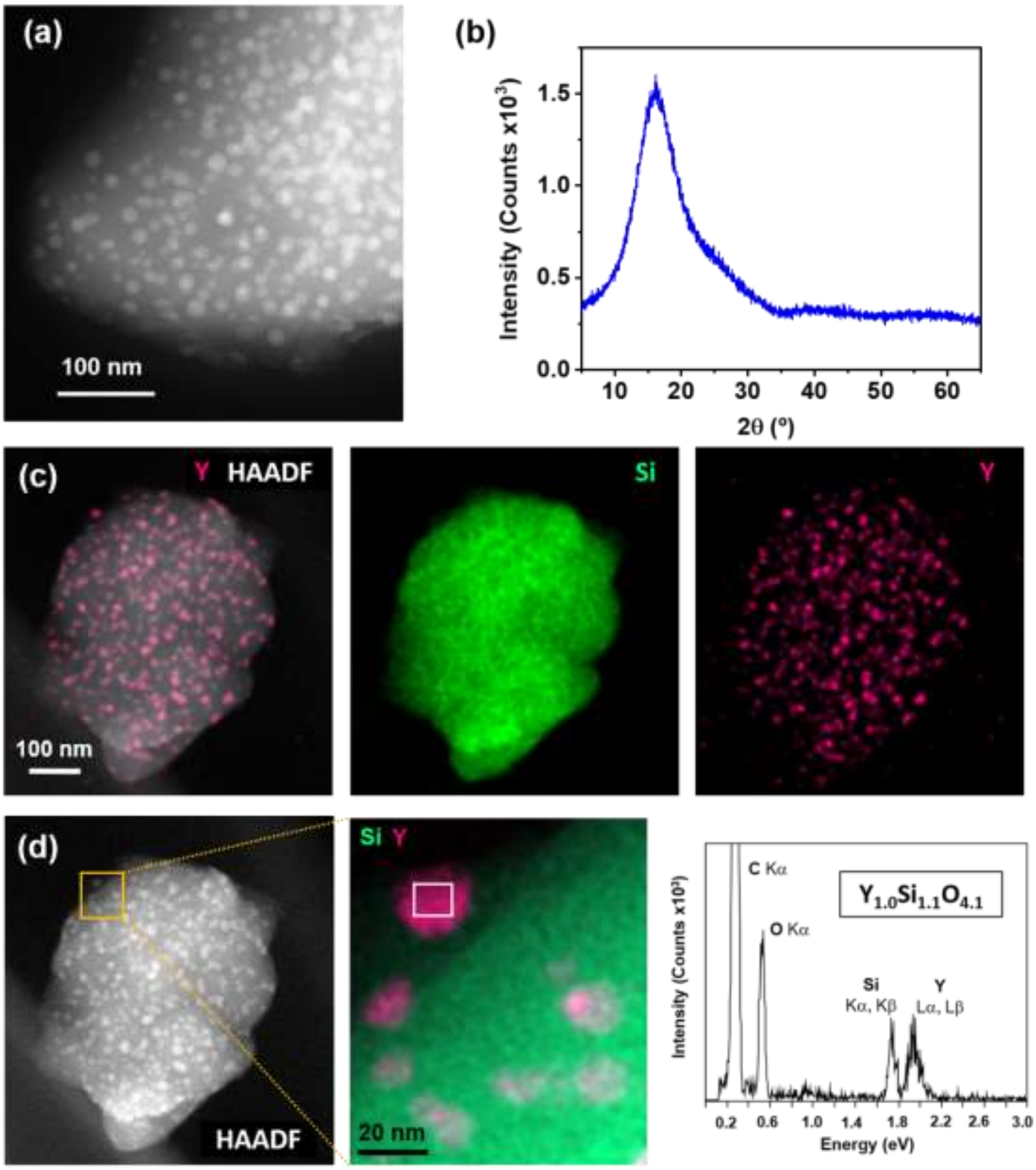

Figure S12. **(a)** HAADF image and XRD pattern **(b)** of the material obtained by annealing for 3h at 1100 ºC a silica gel prepared without iron nitrate and with the same Y:Si molar ratio and solution pH as for the synthesis of sample Y10. **(c)** HAADF image superposed with Y EDX elemental map (left) and Si and Y EDX elemental maps (center and right) of another piece of material showing that the roughly spherical particles of a diameter around 20 nm are composed of Si and Y. O (EDX map not shown) was found everywhere as in the case of Si. **(d)** HAADF image (left) with a zone indicated by the yellow rectangle in which an EDX elemental map was acquired to obtain the superposition of the HAADF image with Y and Si EDX elemental maps (center). From the analysis of the EDX spectrum of the zone centered on a Y silicate particle lying in the thinnest part of the glassy fragment, indicated by a white rectangle; one obtains a composition with a very similar Y to Si atomic ratio (left).

Section 7. TEM and STEM characterization of samples prepared by bulk sol-gel synthesis with La^{3+} and Dy^{3+}

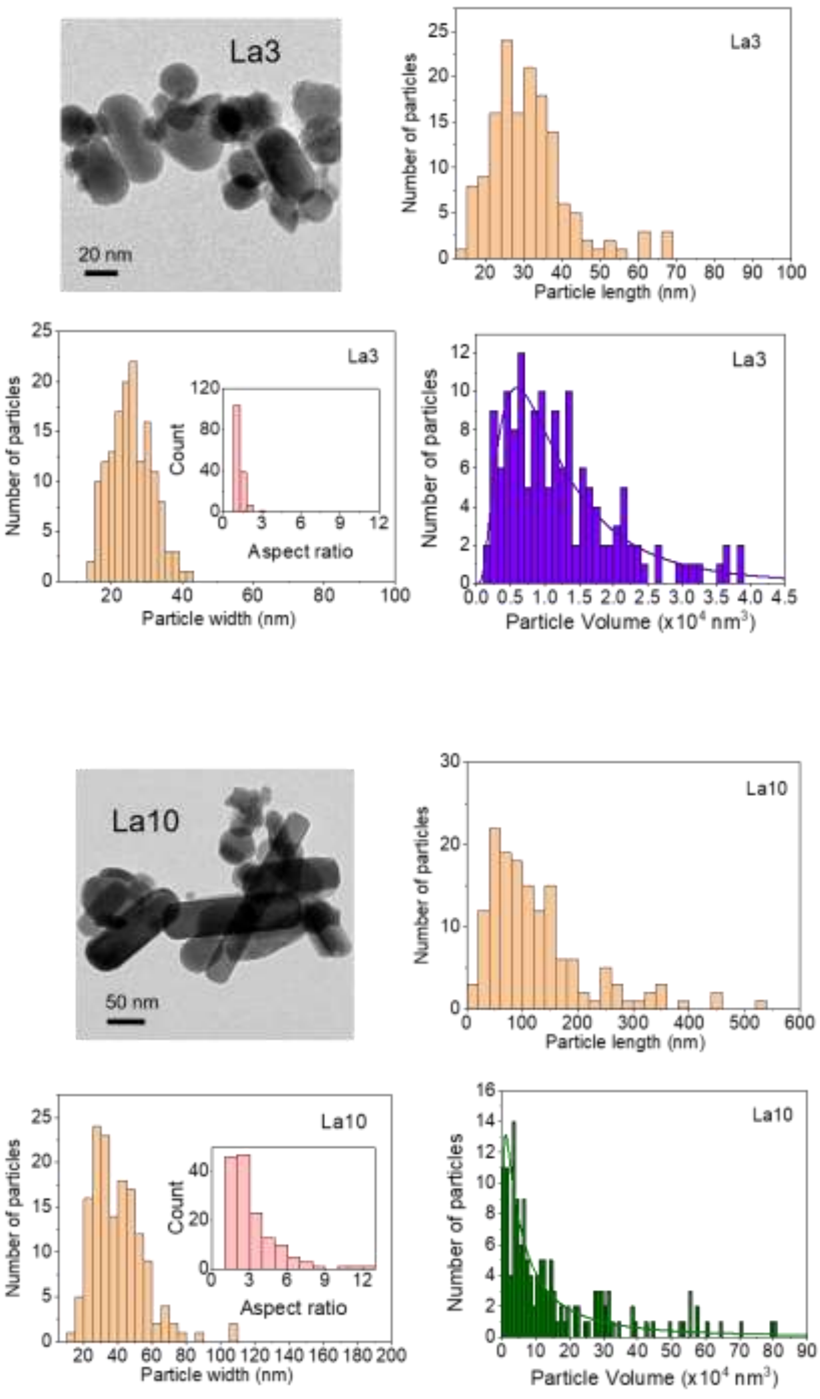

Figure S13. TEM images of ε-Fe_2O_3 nanoparticles prepared different with La^{3+} content *x*= 0.03 (La3) and *x*=0.1 (La10), and the distributions of the lengths, widths, aspect ratios and volumes of nanoparticles.

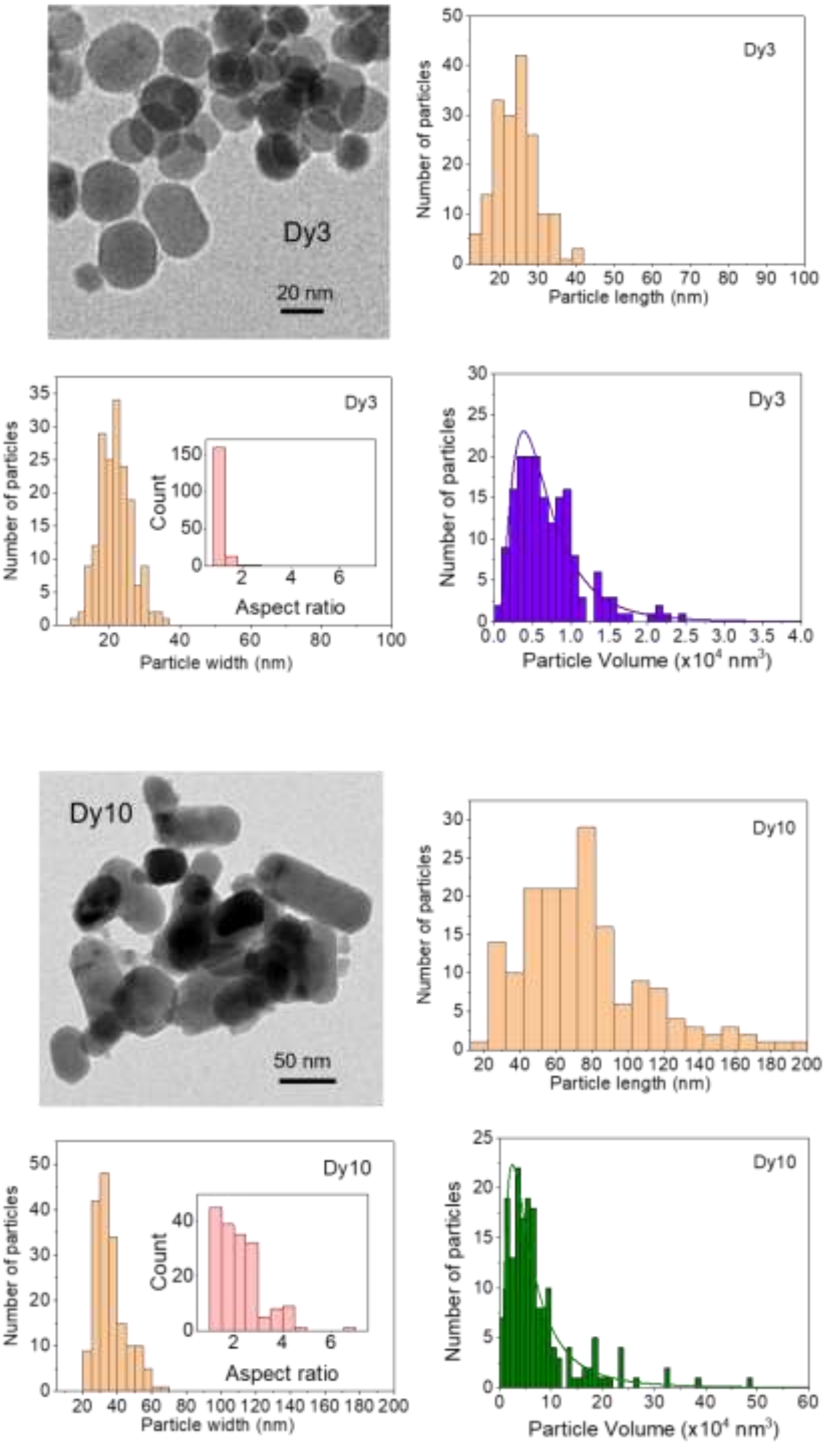

Figure S14. TEM image of ε-Fe_2O_3 nanoparticles prepared with different Dy^{3+} content *x*= 0.03 (Dy3), and *x*=0.1 (Dy10), and the distributions of the lengths, widths, aspect ratios and volumes of nanoparticles.

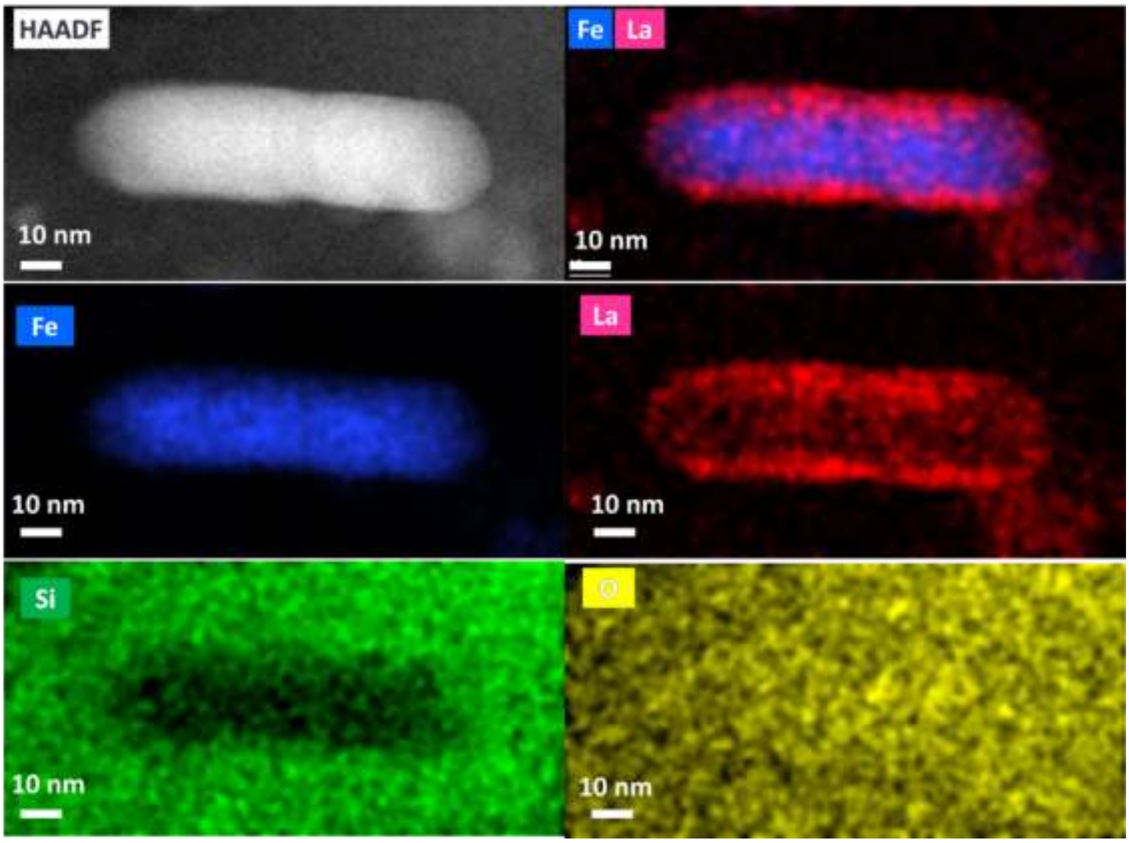

Figure S15. HAADF and EDX elemental maps for a single nanorod inside the silica matrix prepared with a La^{3+} content x= 0.1.

Section 8. Samples prepared with Bi^{3+}, Cd^{3+} and Sb^{3+}

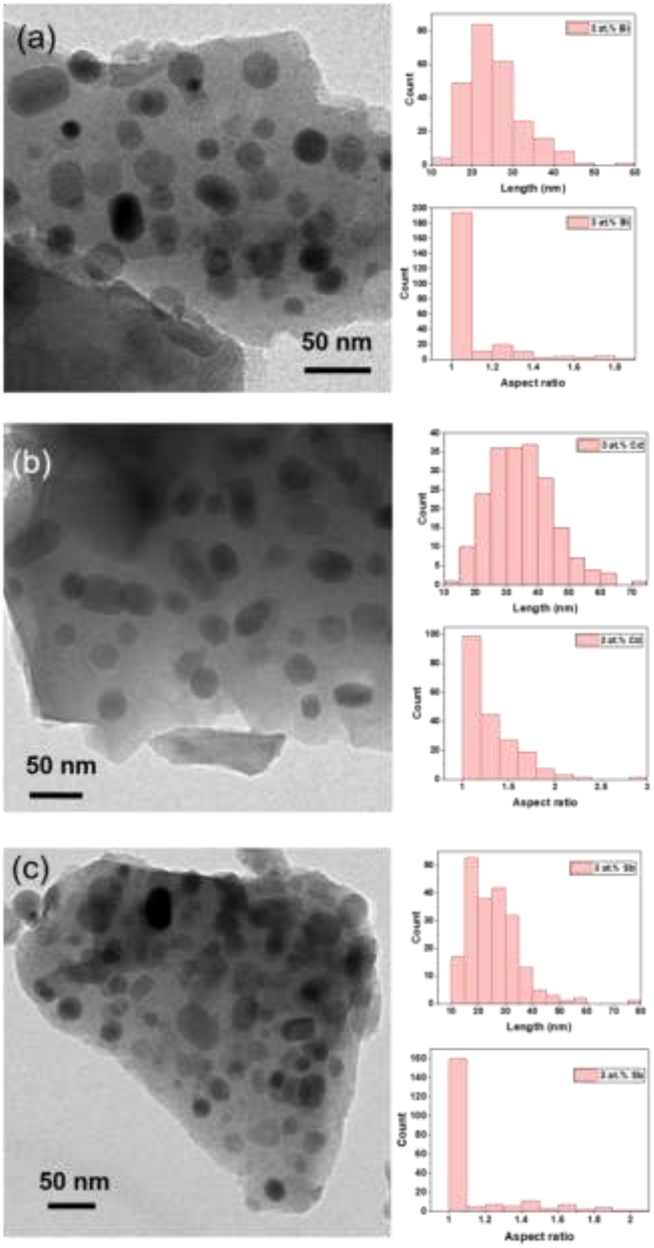

Figure S16. Representative TEM images and length and aspect ratio distributions of non-etched Fe_2O_3/SiO_2 composites prepared with equal concentrations (x=3) of Bi^{3+} **(a)**, Cd^{3+} **(b)** and Sb^{3+} **(c)**. The addition of these cations to the sol also results in an increase of the ε-Fe_2O_3 nanoparticle size but has other consequences such as the crystallization of the amorphous SiO_2 matrix, which induces the formation of hematite, α-Fe_2O_3, (in the case of Bi^{3+}, Cd^{3+}), or the cation substitution of Fe^{3+} into the ε-Fe_2O_3 structure (in the case of Sb^{3+}).

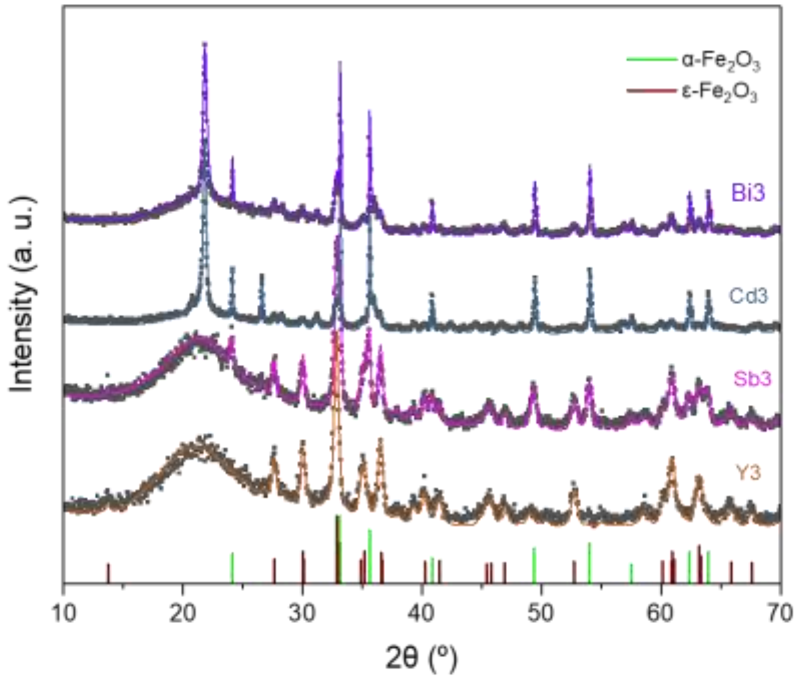

Figure S17. X-ray diffraction of non-etched Fe_2O_3/SiO_2 composites prepared with equal concentrations (*x*=3) of Bi^{3+}, Cd^{3+}, Sb^{3+} and Y^{3+} (sample Y3 in the main manuscript). For the samples containing Bi^{3+} and Cd^{3+} we can observe the partial crystallization of the amorphous SiO_2 matrix, which induces the formation of hematite α-Fe_2O_3. In the case of the sample containing Sb^{3+}, the Rietveld refinement shows an increase in the lattice parameters with respect to the Y3 sample, which indicates some degree of substitution of Fe^{3+} by Sb^{3+}.

Table S6. Lattice parameters of ε-Fe_2O_3

**Sample**	***a* (Å)**	***b* (Å)**	***c* (Å)**
Sb3	5.102(3)	8.809 (2)	9.479(1)
Y3	5.092(8)	8.789(3)	9.473(6)

Section 9. Evolution of nanoparticle sizes with annealing time

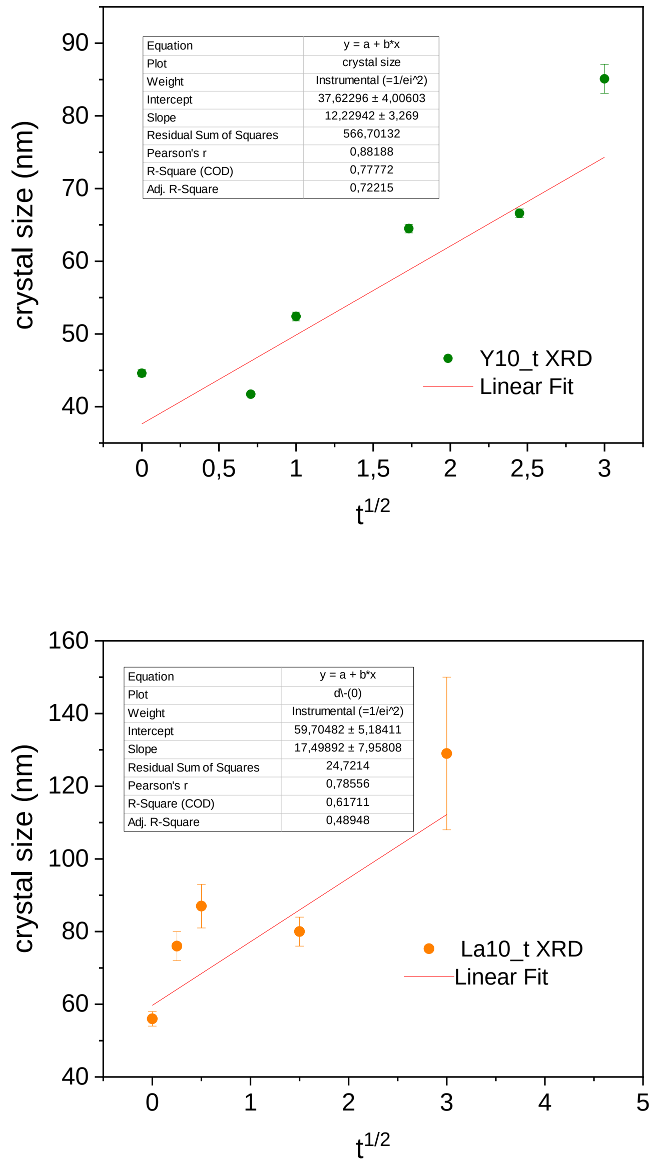

Figure S18. Evolution of the size of ε-Fe_2O_3 nanoparticles obtained from the XRD Rietveld refinement as a function of the square root of annealing time at 1100 ℃. The red lines are linear fits to the data and the table gives the fit results. The goodness of fit parameters indicates that the dependence of the crystal size vs. $t^{1/2}$ is not linear.

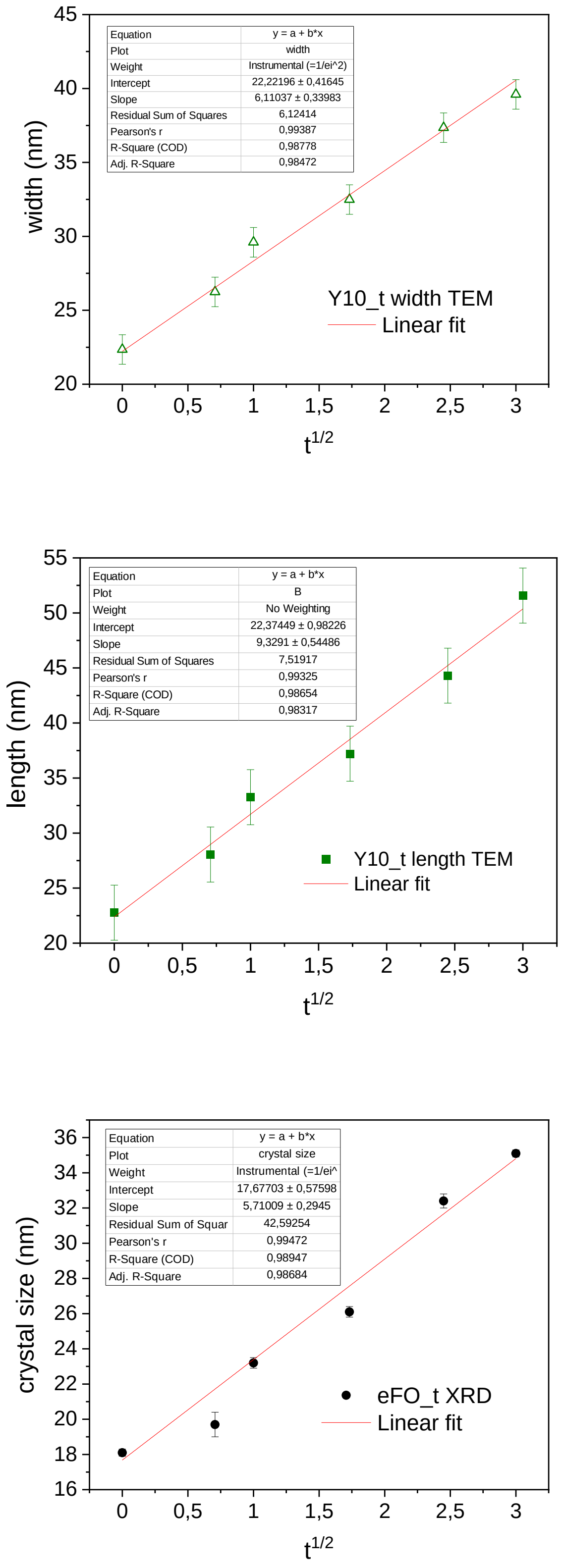

Figure S19. Evolution of the size of ε-Fe_2O_3 nanoparticles obtained from the XRD Rietveld refinement (upper and lower graphs) and from TEM size distributions (middle graph) as a function of the square root of annealing time at 1100 °C. The red lines are linear fits to the data and the table gives the fit results. The goodness of fit parameters indicate that the dependence of the crystal size vs. $t^{1/2}$ is linear.

Section 10. Evolution of nanoparticle sizes with temperature

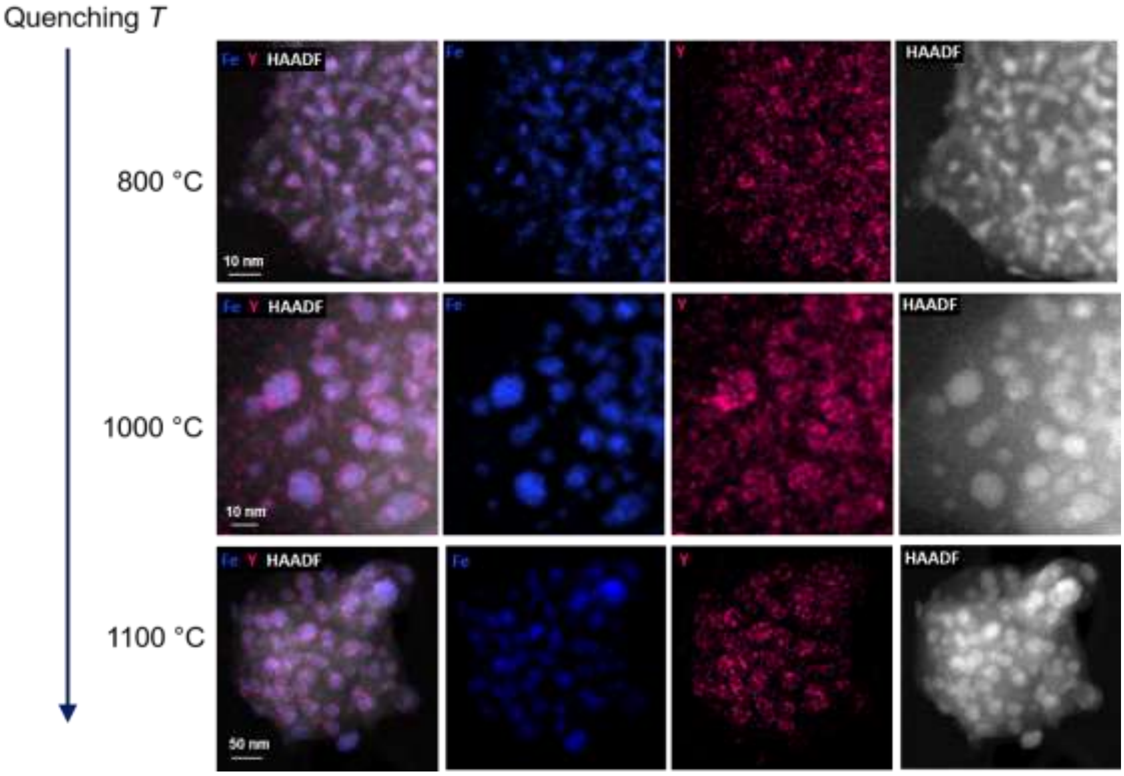

Figure S20. HAADF and EDX elemental maps of ε-Fe_2O_3 nanoparticles inside the silica matrix prepared with Y concentration *x*= 0.1 (Y10) as a function of temperature. The samples were heated at 80ºC/min and quenched from 800ºC, 1000ºC and 1100ºC. Notice that with increasing quenching temperatures there is a significant decrease of the number of particles per unit area and an increase in the average size of the particles, which are both characteristic of a growth process governed by Ostwald ripening. For all the samples, Y is preferentially clustered on the Fe_2O_3 nanoparticles but does not form a continuous layer.

Section 11. STEM characterization of etched Y10 sample prepared by bulk sol-gel synthesis with Y^{3+}

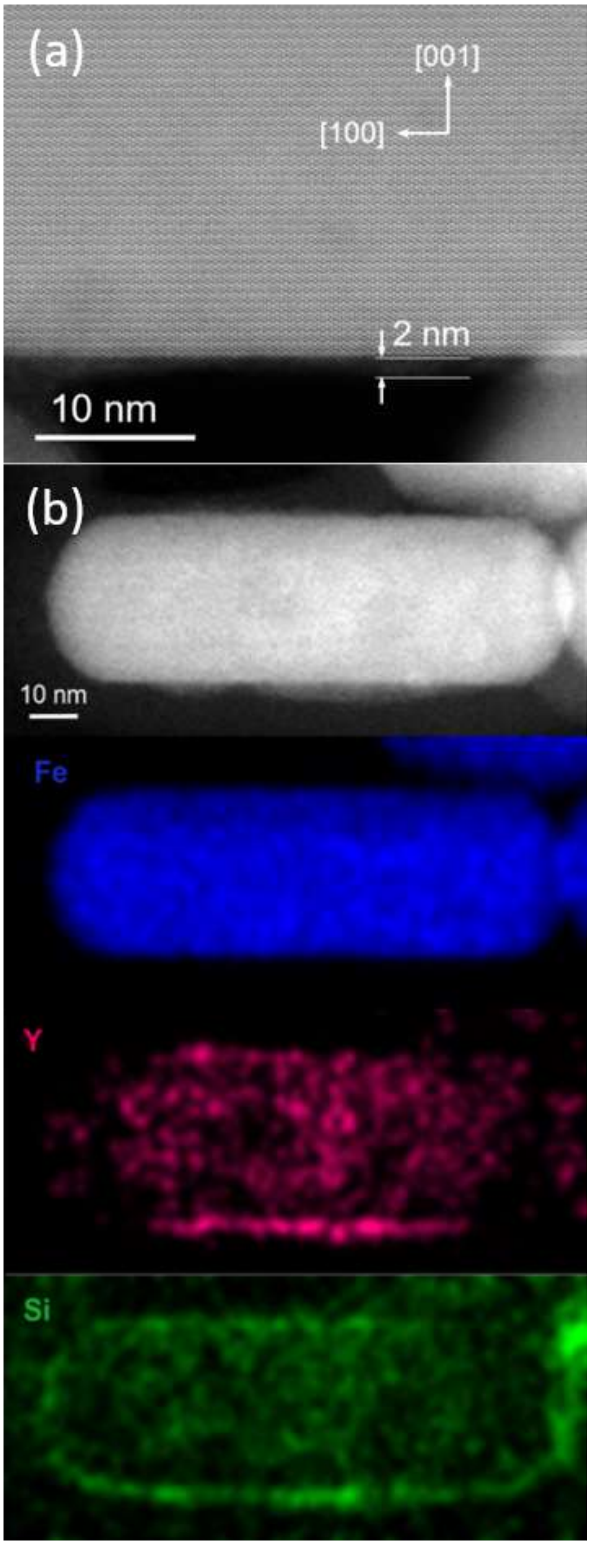

Figure S21. (a) Atomic resolution image of a single nanorod of sample Y10 (*x*=0.1) in a view along the [010] zone axis. It shows that the thickness of the Y silicate layer I sonly of about 2 nm. **(b)** Lower magnification HAADF image of the same nanorod and corresponding elemental maps of Fe, Y and Si, from top to bottom.

Section 11. Magnetization of ε-$(Fe_{1-x}Cr_x)_2O_3$ nanoparticles prepared with Y^{3+}

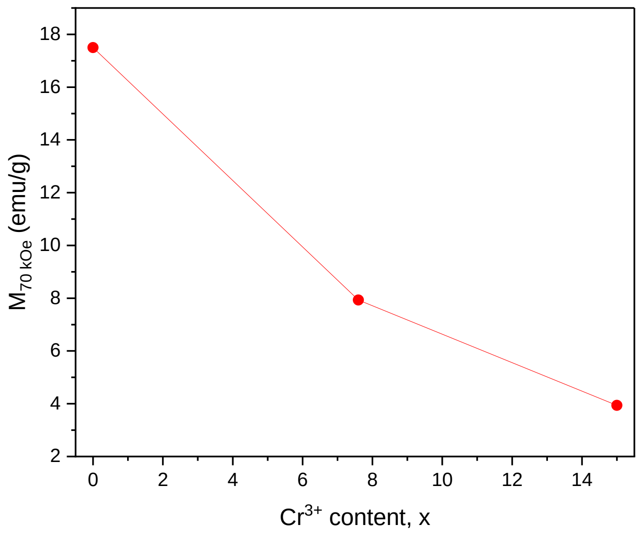

Figure S22. Changes in the magnetization at the highest applied field H=70 kOe as a function of Cr^{3+} content for ε-$(Fe_{1-x}Cr_x)_2O_3$ prepared with Y^{3+} additions.

References

[1] J. Schindelin et al., “Fiji: an open-source platform for biological-image analysis,” Nat. Methods 2012 97, vol. 9, no. 7, pp. 676–682, Jun. 2012.

[2] Maud : L. Lutterotti, S. Matthies and H.-R. Wenk, *IUCr Newsl. CPD*, 1999, 14–15.

[3] A. Jain et al. “The Materials Project: A materials genome approach to accelerating materials innovation” *APL Mater.* **1**, 011002 (2013).

https://doi.org/10.1063/1.4812323

[4] D. P. Kovács et al., “Evaluation of the MACE force field architecture: From medicinal chemistry to materials science”, J. Chem. Phys. 159, 044118 (2023); I. Batatia et al., “A foundation model for atomistic materials chemistry” arXiv preprint DOI: 10.48550/arXiv.2401.00096. ; I. Batatia, D. P. Kovács, G. N. C. Simm, C. Ortner, Gabor Csanyi, “Higher Order Equivariant Message Passing Neural Networks for Fast and Accurate Force Fields” in Advances in Neural Information Processing Systems, editors A. H. Oh, A. Agarwal, D. Belgrave and Kyunghyun Cho, 2022, https://openreview.net/forum?id=YPpSngE-ZU; B. Deng, P. Zhong, K. Jun, J. Riebesell, K. Han, Kevin and C. J. Bartel, Christopher J and G. Ceder, Gerbrand, “CHGNet as a pretrained universal neural network potential for charge-informed atomistic modelling”, Nature Machine Intelligence, 5 (2023) 1031-1041.

[5] H. Larsen *et al* “The atomic simulation environment—a Python library for working with atoms” *J. Phys.: Condens. Matter* **29,** 273002 (2017) **DOI** 10.1088/1361-648X/aa680e

[6] Humphrey, W., Dalke, A. and Schulten, K., "VMD - Visual Molecular Dynamics", J. Molec. Graphics, 1996, vol. 14, pp. 33-38.

[7] Thomas Keating Ltd. (C), https://www.terahertz.co.uk/tk-instruments/products/vectornetworkqo.
